# A State Space Model for Spatial Updating of Remembered Visual Targets during Eye Movements

**DOI:** 10.3389/fnsys.2016.00039

**Published:** 2016-05-12

**Authors:** Yalda Mohsenzadeh, Suryadeep Dash, J. Douglas Crawford

**Affiliations:** ^1^York Center for Vision Research, Canadian Action and Perception Network, York UniversityToronto, ON, Canada; ^2^Department of Physiology and Pharmacology, Robarts Research Institute, Western UniversityLondon, ON, Canada; ^3^Departments of Psychology, Biology, and Kinesiology and Health Sciences, York UniversityToronto, ON, Canada

**Keywords:** spatial updating, predictive remapping, continuous updating, saccade, smooth pursuit

## Abstract

In the oculomotor system, spatial updating is the ability to aim a saccade toward a remembered visual target position despite intervening eye movements. Although this has been the subject of extensive experimental investigation, there is still no unifying theoretical framework to explain the neural mechanism for this phenomenon, and how it influences visual signals in the brain. Here, we propose a unified state-space model (SSM) to account for the dynamics of spatial updating during two types of eye movement; saccades and smooth pursuit. Our proposed model is a non-linear SSM and implemented through a recurrent radial-basis-function neural network in a dual Extended Kalman filter (EKF) structure. The model parameters and internal states (remembered target position) are estimated sequentially using the EKF method. The proposed model replicates two fundamental experimental observations: continuous gaze-centered updating of visual memory-related activity during smooth pursuit, and predictive remapping of visual memory activity before and during saccades. Moreover, our model makes the new prediction that, when uncertainty of input signals is incorporated in the model, neural population activity and receptive fields expand just before and during saccades. These results suggest that visual remapping and motor updating are part of a common visuomotor mechanism, and that subjective perceptual constancy arises in part from training the visual system on motor tasks.

## 1. Introduction

Spatial updating is the ability to locate targets that were perceived before an intervening self-motion displaces the original sensory frame of reference (Baker et al., 2003; Klier and Angelaki, 2008; Sommer and Wurtz, 2008; Crawford et al., 2011). In the oculomotor system, this is often studied using the “double step” task, where subjects are required to make an accurate saccade toward a remembered visual target after an intervening eye motion. Such experiments have shown that humans and monkeys are able to do this despite intervening saccades (Hallett and Lightstone, 1976; Mays and Sparks, 1980), head-unrestrained gaze shifts (Munoz et al., 1991), smooth pursuit eye movements (Lisberger et al., 1987; Schlag et al., 1990; Herter and Guitton, 1998; Baker et al., 2003; Medendorp et al., 2003; Blohm et al., 2005; Klier and Angelaki, 2008), translational motion of the head/body (Angelaki and Yakusheva, 2009; Clemens et al., 2012), and torsional rotation of the head/body (Medendorp et al., 2003; Klier and Angelaki, 2008). The detailed neurophysiological mechanisms are not yet known, but it is thought that this involves the use of internal efference copies of eye motion (Mays and Sparks, 1980; Sommer and Wurtz, 2006), and results in the recalculation of an appropriate motor saccade vector for the final eye position in areas such as the superior colliculus (SC) (Groh and Sparks, 1996).

Spatial updating in the oculomotor system may be intimately related to visual space constancy—perception of constant spatial locations despite frequent displacement of the retina by saccades—and may share common mechanisms (Wurtz, 2008). The neurophysiology of visual space constancy has mainly been studied using the single-step saccade task, where a task-irrelevant stimulus is presented just before a saccade. This often results in the phenomenon known as predictive remapping, which has been observed through higher level visual and visuomotor areas of the brain (Duhamel et al., 1992; Walker et al., 1995; Umeno and Goldberg, 1997; Nakamura and Colby, 2002; Sommer and Wurtz, 2006). Neurons that show predictive remapping are activated by saccades that will bring visual stimulus into their receptive fields (RFs) even before the actual movement of the eyes (Duhamel et al., 1992). This is often interpreted in terms of the visual properties of single neurons, i.e., as a transient extension of the RF toward the future location of the stimulus; the future RF. However, some neurons that show predictive remapping also show sustained activity at the new eye position when the standard RF stabilizes over the previous stimulus location (Duhamel et al., 1992). Further, both of these phenomena can be explained as equivalent to the trans-saccadic transfer of activity from the original population of neurons representing the visual stimulus relative to initial gaze position to another population coding for the stimuli relative to the final gaze position (Keith and Crawford, 2008). This retained gaze-centered information could then be used for a variety of purposes, including saccade generation. Thus, remapping, spatial memory, and sensorimotor updating may involve several common or inter-related features (Mays and Sparks, 1980; Duhamel et al., 1992; Walker et al., 1995; Batista et al., 1999; Tian et al., 2000; Nakamura and Colby, 2002; Balan and Ferrera, 2003a,b).

There is also reason to expect somewhat different mechanisms for updating visual space during behaviors that involve slow, continuous motion of the eye in space, like smooth pursuit eye movements or translations of the head (Angelaki and Yakusheva, 2009; Clemens et al., 2012; Dash et al., 2015). First, unlike saccades, slow, continuous eye movements often have unpredictable trajectories. Second, unlike saccades, slower eye movements do not suppress vision, presumably because the retina still provides useful information and thus it is useful and important to maintain vision during longer-duration eye motion. For such movements predictive remapping is not ideal, but rather spatial updating should occur continuously. This prediction was recently confirmed by recording from neurons in the SC during a double-step smooth pursuit-saccade task. In this experiment most visual cells showed gaze-centered memory-related activity for the saccade target, such that the population showed a “moving hill” of activity across the SC topographic map during pursuit (Dash et al., 2015). Here, visual memory activity was spatially-specific, only occurring when the remembered target crossed the visual RF, and motor activity only occurred at the end of pursuit, just before the saccade.

Despite numerous investigations on this topic, many unanswered questions remain about the theory and mechanisms of visual and visuomotor updating (Thier and Ilg, 2005; Ibbotson and Krekelberg, 2011). Since experimental work is extremely difficult and often (in animal experiments) can only target selected areas and signals at one time, it is important to have a theoretical framework to guide such experiments. Past theoretical efforts have used control-system type models to explain the spatiotemporal and geometric aspects of updating (Quaia et al., 1998; Optican and Quaia, 2002; Blohm et al., 2006; Cromer and Waitzman, 2006; Van Pelt and Medendorp, 2007), and neural network models to predict specific signals (Zipser and Andersen, 1988; White and Snyder, 2004, 2007; Keith et al., 2010). However, there is still no general theoretical framework for spatial updating and remapping. Here, we took a step in this direction by examining whether training on the motor aspects of spatial updating can produce the updating/remapping signals that have been observed in visual neurons. To do this, we developed a state-space model (SSM) for updating target-related spatial information in gaze-centered coordinates. SSMs provide an effective method for modeling dynamical systems and it can represent the internal behavior of these systems.

Here, we constrained our model to receive simulated inputs (Visual RFs, eye positions signals, and eye movements signals) that have already been physiologically verified (Walker et al., 1995; Hanes and Schall, 1996; Sommer and Wurtz, 2002; Marino et al., 2008; Morris et al., 2012), and trained it to update the location of saccade target after an intervening saccade or smooth pursuit movement. After training, the RFs of state-space units replicated both predictive remapping during saccades (Duhamel et al., 1992) and continuous eye-centered updating during smooth pursuit (Dash et al., 2015). In addition, during trans-saccadic remapping, RFs also expanded (a prediction which to our knowledge has not yet been reported in the published literature). These findings demonstrate that, in principle, the neural phenomena associated with the remapping of visual responses can arise from training on a motor task, and thus suggest a strong association between visual remapping and motor updating.

## 2. Materials and methods

The proposed model aims to study the dynamics of spatial updating through time in two types of eye movements, saccades and smooth pursuits. As in previous models on this general topic (Zipser and Andersen, 1988; White and Snyder, 2004, 2007; Keith et al., 2010) we aimed to model this system at a level that bridges the computational and algorithmic levels (Marr, 1982), and made no attempt to model mechanisms at the biophysical level. In order to simulate the dynamics of neural mechanism during smooth pursuit and saccades, we used these novel approaches: we developed a SSM and we used a dual Extended Kalman filter (DEKF) approach (Wan and Nelson, 1997, 2001). More specifically, we used two interleaved Extended Kalman filters (EKFs), (1) one for state estimation (signal estimation) and (2) the other one for weights estimation (model estimation). In our proposed model, we tried to not only replicate the behavioral aspect of updating but also show the neural mechanism underlying this phenomenon. In this section, we first explain the task widely used to study spatial updating and then explain our proposed model intuitively in a simple way.

### 2.1. Double-step tasks

**Figure 1:**
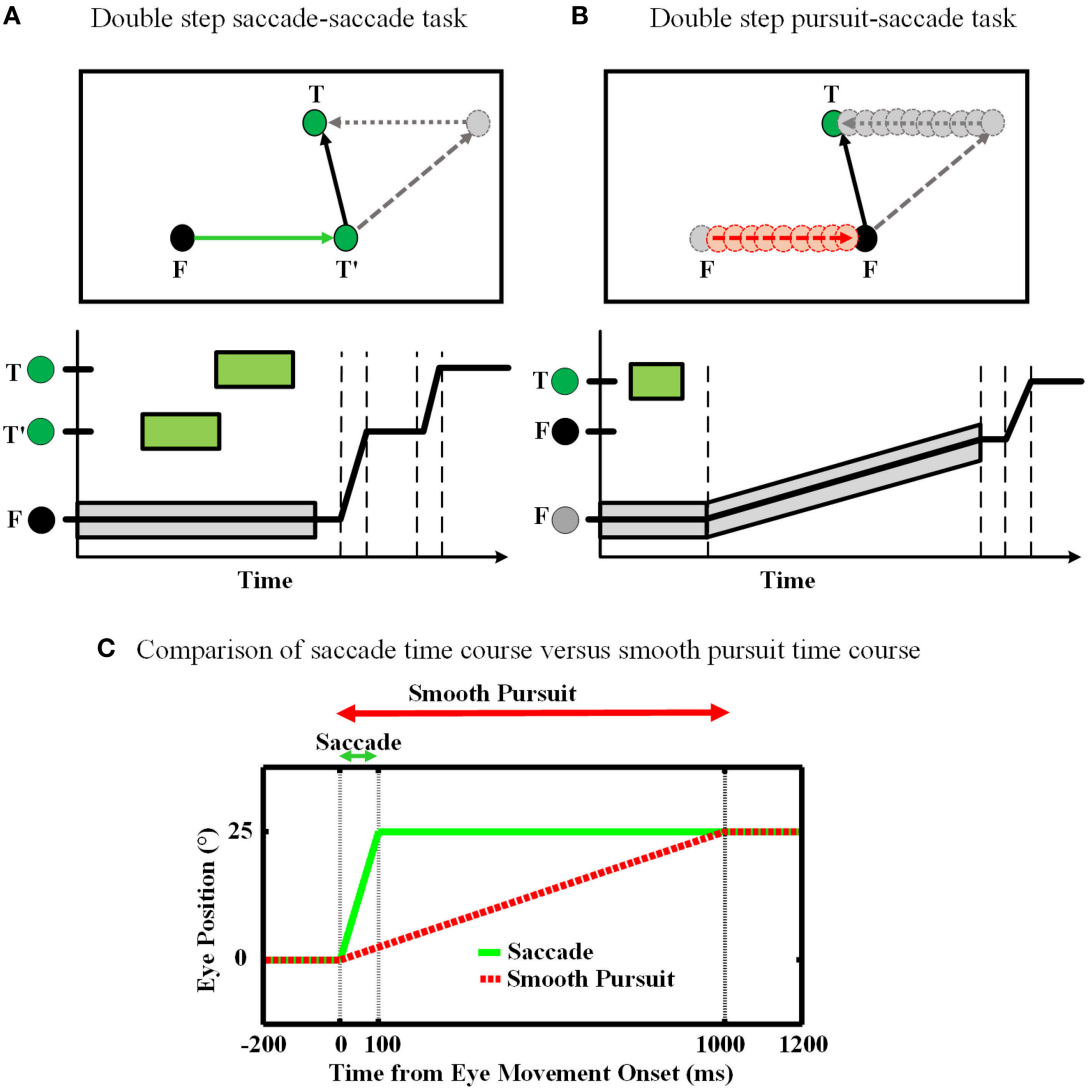
**Spatial updating of second target position in retinotopic coordinates. (A)** Spatial updating of remembered second target position during a saccadic eye movement. Upper panel shows the configuration of the double step saccade-saccade task in 2D spatial coordinates. When the subject is fixating to the fixation point F (black circle), the locations of the two visual targets T′ and T (green circles) are coded in retinotopic coordinates relative to F. After the completion of the first intervening saccade to T′, if spatial updating were not occurring the gray circle would be the destination of the second saccade. But primates are able to compensate for the first saccade (dotted arrow) and update the location of T relative to their new gaze position (T′) and thus make a correct saccade to the position of T. The lower panel shows the eye position in time during the saccade-saccade task. The timing of the visual targets appearance is depicted by green rectangular which shows the duration of each target presentation. Also the gray rectangular shows the duration of the fixation point appearance. **(B)** Spatial updating of remembered second target location during a slow continuous eye movement. While subject is fixating to the fixation point F, the position of the remembered target T is coded in retinotopic coordinates relative to F. During the smooth pursuit phase, while the subject is following the slowly moving fixation point (red dashed arrow), the position of the previously perceived target T is getting updated continuously and gradually (gray dotted arrow) as the eyes are moving. Therefore, in the end of pursuit the subject can make a saccade to the correct position of T. The lower panel shows the eye position in time during smooth-saccade task. The green rectangular shows the duration of the target presentation. The timing of the fixation point presentation is shown by a gray shadow around the position curve which also shows the slow movement of the fixation point. **(C)** Comparison of these two tasks in terms of timing. The duration of a 10° saccade is around 50 ms which is much less than a typical smooth pursuit which can be around 1000 ms.

The double step task is a common approach to study the concept of spatial updating. In our work, we consider two forms of this task: (1) saccade-saccade (Hallett and Lightstone, 1976; Mays and Sparks, 1980), and (2) pursuit-saccade (Schlag et al., 1990; Herter and Guitton, 1998; Baker et al., 2003; Medendorp et al., 2003; Blohm et al., 2005; Klier and Angelaki, 2008). Figure 1 illustrates the spatial geometry of these tasks (A and B), and compares their relative timing. In the saccade-saccade task (Figure 1A) subjects fixate (F) while a target (T′) briefly appears (and then disappears) in the visual periphery. Then a second target (T) briefly appears. When the fixation point (F) disappears, the subject must make two successive saccades to the remembered position of T′ and then T. The pursuit-saccade task (Figure 1B), is similar, except that there is only one saccade target (T) and after it disappears, F starts to slowly move. The subject must follow F until it disappears. At that time, the subject makes a saccade to the remembered position of the previously shown target T.

Smooth pursuit differs from saccades in several fundamental ways (Figure 1C). First, saccades are ballistic: they are very brief and rapid (often ≥ 500 degree/s) and thus do not receive or require visual feedback (Straube et al., 1997). They are internally driven by a discrete burst of activity and obey lawful behavioral relationships (Robinson, 1964; Fuchs and Luschei, 1970). This provides the necessary conditions for a mechanism that predicts both saccade duration and final eye position (Duhamel et al., 1992; Walker et al., 1995; Umeno and Goldberg, 1997; Nakamura and Colby, 2002; Sommer and Wurtz, 2006). In contrast, pursuit requires visual feedback based on movement of the fixation point, which may be unpredictable in speed, length, and duration. However, pursuit is also slower (≤ 100 degree/s), allowing ample time for on-line feedback not only from retinal signals, but also extra-retinal signals such as an accurate sense of current eye position and velocity. This provides the necessary conditions for a mechanism that continuously updates remembered target position.

Humans and animals are able to perform both of these two tasks almost accurately in the dark, i.e., based on egocentric cues in the absence of allocentric cues (Klier and Angelaki, 2008; Sommer and Wurtz, 2008). This means that the final saccade target (T) must be stored and updated within some egocentric frame of reference. Otherwise, if subjects simply used the original visual vector for T, they would generate the wrong saccade (gray dashed lines in Figures 1A,B). Most experimental evidence suggests that visual targets like T′ and T are coded in short-term memory using gaze-centered coordinates (Walker et al., 1995; Sommer and Wurtz, 2002; Marino et al., 2008). As a result, there has to be a mechanism in the brain which updates the remembered position of T′ in spite of the intervening changes in the gaze position during the first eye movement. In effect, the original visual vector (gray dashed vectors) in Figures 1A,B must be transformed by the reverse of the first eye movement vector (gray dotted lines) into the correct second saccade vector (black solid lines). In the current study, we aimed to simulate both the behavior and emergent neural mechanisms associated with spatial updating during the pursuit-saccade and saccade-saccade tasks, using the model described in the next subsection.

### 2.2. Model structure

**Figure 2:**
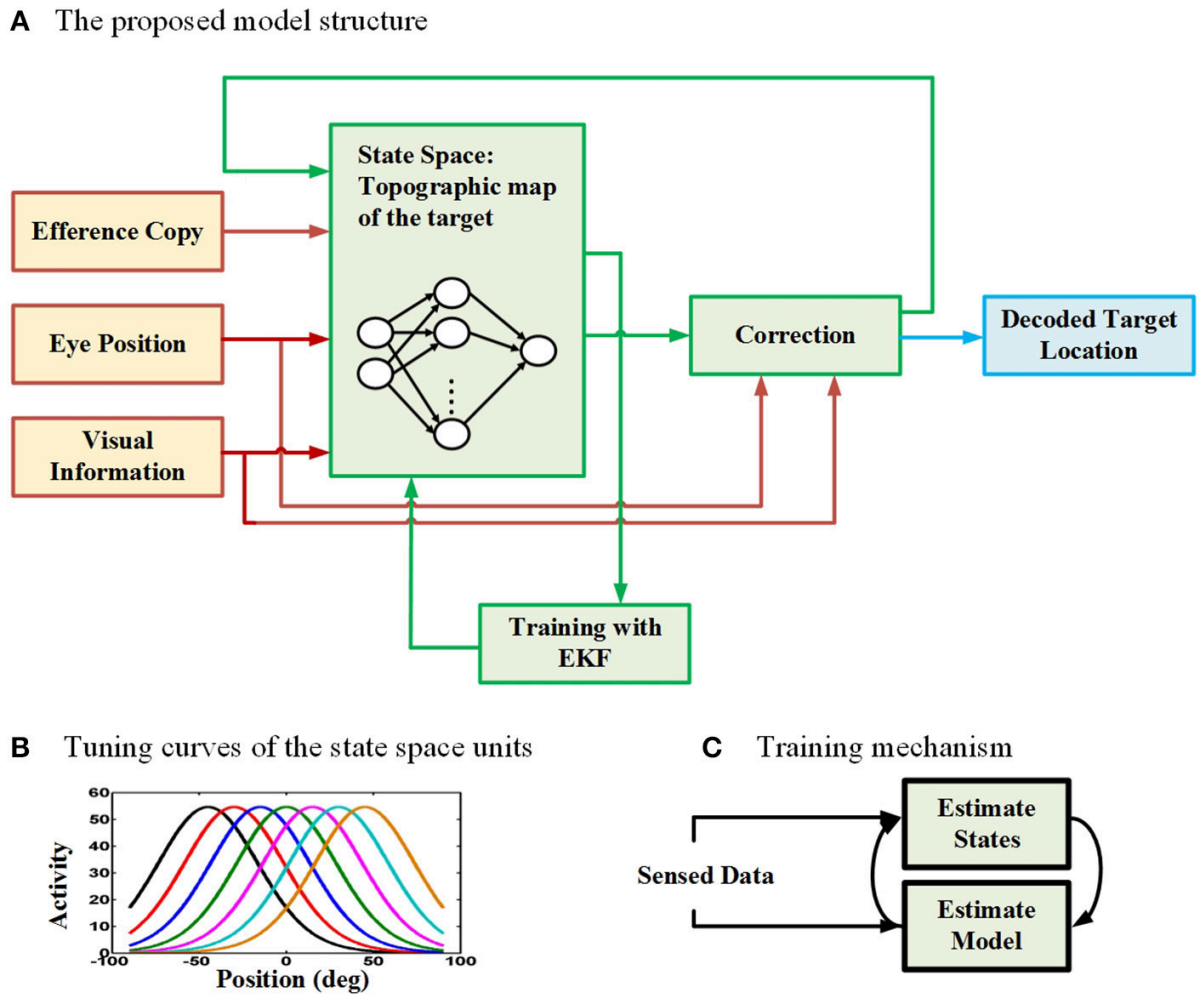
**The proposed model for spatial updating during eye movements. (A)** The architecture of the proposed model which takes eye position, efference copy, and visual information as input signals (The input connections and boxes are indicated with red color arrows) and it produces the decoded location of the remembered target through time as output (The output connection and box are indicated with blue color). A three layer neural network is considered to model the state space units (The internal connections of the model are illustrated in green). **(B)** The tuning curves of the state space units in the hidden layer of the neural network are spatially selective radial basis functions in gaze-centered coordinates. **(C)** The training mechanism in the proposed model. The model uses an expectation maximization approach to estimate both the model parameters (parameters of the neural network) as well as the states (position of the second target).

To model the neural behavior of the brain in spatial updating paradigm, we developed a recurrent radial basis function neural network (RBFNN) trained with an EKF method (Wan and Nelson, 1997, 2001; Huang et al., 2005; Vukovi and Miljkovi, 2013). Figure 2 demonstrates the general structure of our proposed model. The proposed model aims to update the location of a perceived target given that an eye movement is occurring. The main part of the model is the box consisting of a three-layer neural network. The input layer takes three types of input (red lines): (1) an efference copy signal (depending on the task, this signal is a motor burst signal or an eye velocity signal), (2) an eye position signal, and (3) visual target information. The input layer receives the input signals and then distributes them through a full connection to an intermediate layer. As we mentioned before, the model aims to keep track of the second target location during the saccade-saccade task or the remembered saccade target position in the pursuit-saccade task. This target position is encoded in the brain in a gaze-centered population code. This target position is an internally encoded signal and cannot be sensed or measured directly. This population of neurons encoding target position in gaze-centered coordinates constructs the state space in our model and it is developed by a layer of radial basis function (RBF) units (intermediate layer in Figure 2A). Each neuron in this layer has a bell-shaped tuning curve with a specific preferred position (Figure 2B). This RBF layer receives input signals from input layer of the neural network. The output layer produces the inferred location of the second target based on the activity of neurons in the intermediate layer. This prediction is corrected using the input signals and provides a feedback to the input layer of the neural network (a recurrent architecture). The output of the correction box is the updated location of the second target after the first intervening eye movement. This inferred updated position can be used by the brain to send a command for making the second eye movement (saccade) in both types of double step task. Importantly, the model was designed and trained in such a way that it could update remembered targets from one trial to the next in either task (pursuit-saccade and saccade-saccade) using the same set of internal parameters (see Mathematical formalization and Supplementary materials for details). In the following sections we explain the model input signals, training procedure, and mathematical formalization with more detail.

### 2.3. Inputs

**Figure 3:**
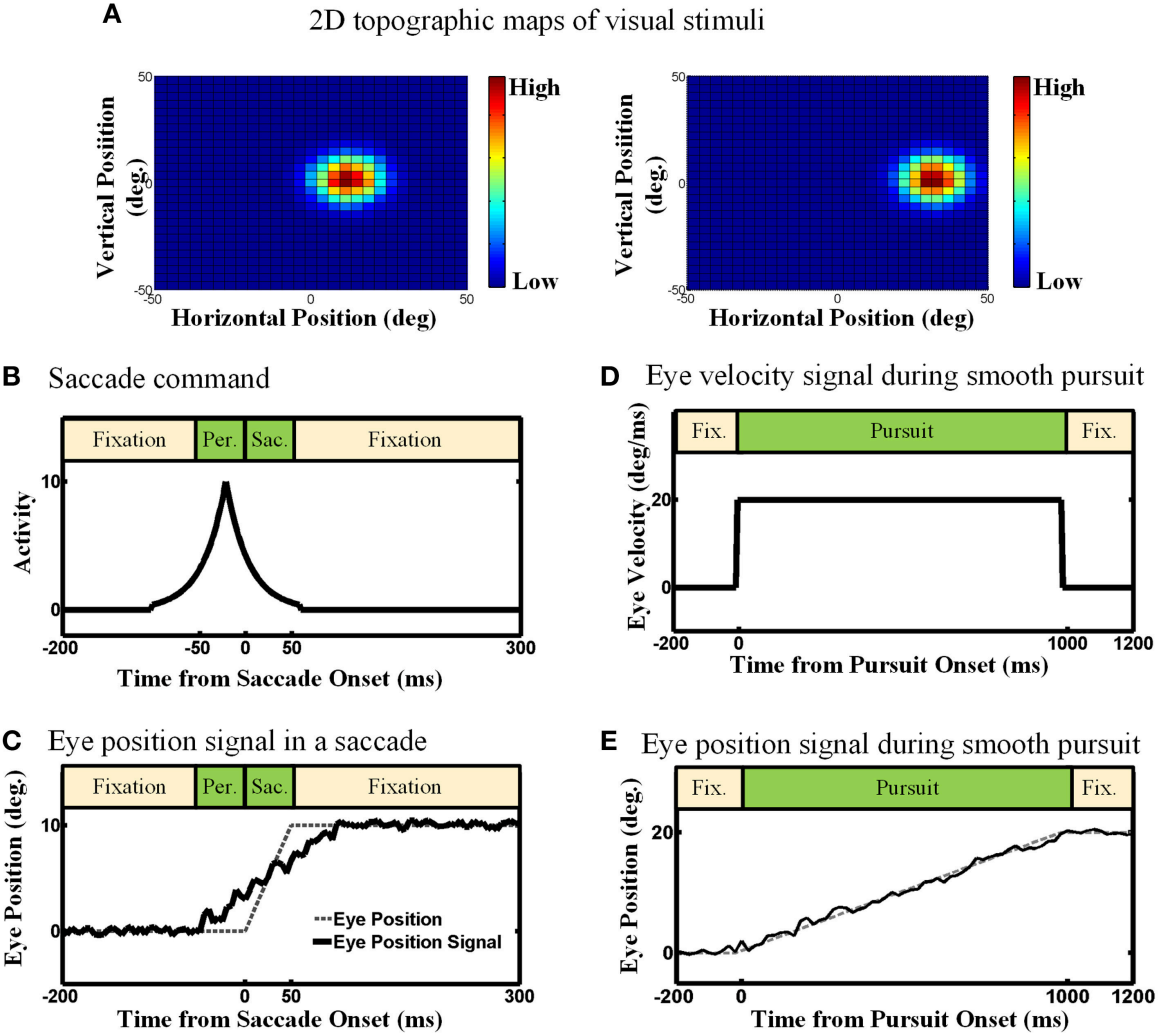
**The model inputs (Examples). (A)** 2D topographic maps of visual stimuli. **(B)** Efference copy signal for the saccadic eye movements. The peak of this signal depends on the size of the saccade. Also the peak occurs before the saccade onset. **(C)** Eye position signal for the saccadic eye movement. This eye position signal can be found in many brain areas like LIP, VIP, MT, and MST. It leads and lags the saccade duration. **(D)** Eye velocity signal in the smooth pursuit eye movement. For simplicity, this Eye velocity is modeled as constant during the smooth pursuit because the fixation point is moving with a constant velocity. **(E)** Eye position signal in the smooth pursuit eye movement. Since the eyes perfectly track the fixation point in our simulation, the eye position signal exactly follows the position of fixation.

We considered three types of input in our proposed model: (1) an efference copy signal, depending on the type of eye movement, it can be a signal inspired by the motor burst in the SC for saccades, or an eye velocity signal for smooth pursuit^1^, (2) an eye position signal, found in the lateral intraparietal cortex (LIP), the ventral intraparietal (VIP), the middle temporal (MT) and the medial superior temporal (MST) areas (Morris et al., 2012), and (3) visual topographic maps of visual stimuli located in the SC (Walker et al., 1995; Sommer and Wurtz, 2002; Marino et al., 2008). Figure 3 depicts the detailed nature and physiological inspiration of the input signals we employed in our computational model through some examples. Consider a double step task, visual information is the first input that the subject receives and it is encoded as a topographic map in the population activity of midbrain SC neurons as depicted in Figure 3A (Mays and Sparks, 1980; Sparks and Porter, 1983; Walker et al., 1995; Sommer and Wurtz, 2002; Marino et al., 2008). In other words, the visual input is used to initialize the neurons in the state space. Note that, like most previous models of spatial updating (e.g., Keith et al., 2010), we used a homogenous retinotopic map in our model, which is a simplification of the actual SC map (Cynader and Berman, 1972; Munoz and Wurtz, 1993a,b). This simplification reduced the computational complexity of the model without interfering with its ability to simulate spatial updating (see Section 3). Eye position signal is another signal which we used as input in our model. For simplicity and clarity, we illustrate these input signals in one dimension. Depending on the type of the double step task (pursuit-saccade or saccade-saccade), we employed different eye position signal to train our model. For the double-step saccade-saccade task, we employed the eye position signal which can be found in many neurons in LIP, VIP, MT, and MST. As explained in Morris et al. (2012), the main feature of this signal is that it leads the actual eye movement (shown as dashed line in Figure 3C) and lags as the saccade completes. Figure 3C depicts an eye position signal (solid curve) for a 10-degree saccade which lasts for 50 ms. We considered a 50 ms duration for a 10-degree saccade and a 100 ms duration for a 25-degree saccade and we calculated the duration for the other saccade sizes based on a linear relation according to these values (Carpenter, 1988). In the double-step pursuit-saccade task, the subject's sensorimotor system must have an accurate representation of eye position (Noda and Warabi, 1982; Squatrito and Maioli, 1997; Tanaka and Fukushima, 1998); moreover, the eye movement lasts for around 1000 ms. Therefore, we employed an eye position signal as depicted in Figure 3E. This figure shows an eye movement of 20 degrees in 1000 ms (dashed line) and the corresponding eye position signal (solid line) which can be found in many neurons in MT, MST, LIP, and VIP (Bremmer et al., 1997a,b) and it is produced by adding noise to the actual eye position (noise free signal as shown in dash line in Figure 3E). The duration of a smooth pursuit eye movement in time is much longer compared to a saccadic eye movement. The third input is an efference copy signal which is believed to have the major effect on the spatial updating in the brain. This signal also depends on the task. In the pursuit-saccade task, since the brain must have an accurate representation of eye velocity, we used this signal (Figure 3D) as efference copy to train our model. In the saccade-saccade task which involves jerky rapid eye movements, the intended eye displacement in the form of a motor burst signal which can be found in SC (Hanes and Schall, 1996) is used as the efference copy signal (Figure 3B). The peak height of this signal determines the intended eye displacement and as it is depicted in Figure 3B, it leads the saccade onset as it is an efference copy signal.

### 2.4. Training procedure

The proposed model aims to update the stored location of a perceived visual target, knowing that an eye movement is occurring. To this end, the model finds the retinotopic location of the desired target at each time step by integrating the issued eference copy command and the target location at the previous time step. The intermediate layer of the proposed network is initialized with the retinotopic topographic population code in SC. Then the activity of neurons in this layer is estimated through time incorporating the EKF approach. Using the estimated population activities, the model infers the gaze-centered location of the target using another Kalman filter estimation method and this estimation is fed back to the input layer through the recurrent connection and used for the estimation of the population activities in the next time step. In other words, the proposed recurrent neural network computes on-line estimates of the target location as well as population activities through time. We used a dual estimation approach which alternates between estimating the model parameters based on the estimated denoised states and estimating the states using the current model parameters (Figure 2C). The details about convergence of this approach can be found in Wan and Nelson (2001). Next section provides a brief mathematical formalization of the proposed model. Those who are not interested in computational details can skip the next section.

### 2.5. Mathematical formalization

#### 2.5.1. A linear state space model

In this section, we present the details of our proposed model for spatial updating of remembered visual targets across saccadic and smooth pursuit eye movements. This model aims at studying the dynamics of spatial updating and describe the effect of eye position uncertainty in this dynamics. The model which is presented in this section can be considered as the first level in Marr's levels of analysis (Marr, 1982) and it is designed to answer to questions about the effects of uncertainty in the spatial updating phenomenon in system level. We express the problem of spatial updating in double-step task as aiming at estimating the current location of the second target in a gaze-centered frame of reference based on the previous estimation of this location and the efference copy signal about the eye movement. Therefore, we can express the dynamics of spatial updating with the following equation:

(1) $$TR_{k\;+\;1}=TR_{k}-EC_{k}+\nu_{k}$$

where *TR*_*k*_ is a D-dimensional vector representing the second target (memory target) position in the retinotopic frame of reference, *EC*_*k*_ denotes the efference copy signal which describes the eye movement; for saccadic eye movement it is a motor burst-like signal which shows the intended eye movement while for smooth pursuit eye movement it is an eye velocity signal describing the constant velocity of slow motion of the eyes. Of course, there is an error in any estimation; the error associated with this estimation is captured by a zero mean additive noise ν_*k*_ with a variance of $\sigma_{TR}^{2}$. Studying saccadic over/undershoots are out of the scope of the current work; however, over/undershoots can be considered by adding an offset term to Equation (1). As the literature suggests this offset is dependant to the target distance (Deuble et al., 1984; Becker, 1989; Harris, 1995). Therefore, this offset can be modeled as another input in Equation (1).

The Brain has access to different sources of observations which can be employed to improve the estimation described in Equation (1). Eye position signal which has been found in many brain areas like LIP, VIP, MT, and MST (Morris et al., 2012) seems like the most related noisy observations available in the brain. This signal can be related to the location of the memory target with the following equation:

(2) $$EP_{k}=-TR_{k}+TS+n_{k}$$

where *EP*_*k*_ shows the eye position signal at time point *k*, *TS* denotes the position of the memory target in the head frame of reference, and $n_{k}\sim N\left(0,\sigma_{EP}^{2}\right)$ is an additive observation noise featuring the uncertainty about the eye position signal in the brain. *TS* is required because eye position is in head frame of reference and *TR* is in eye frame of reference and mathematically to write an equation that relates *TR* to *EP*, we need this information to simplify the calculation in a common reference frame, but previous neural network studies have shown that such reference frame transformations can be performed without ever having an explicit representation of space in head or space coordinates (Smith and Crawford, 2005; Blohm et al., 2009).

Equations (1) and (2) form the SSM for our variable of interest *TR*. Please note that Equation (2) provides a relation between the available noisy observation *EP* and the variable of interest *TR*. The aim is to find an optimal estimation of *TR* using both the available observations *EP* and the dynamics described in (1). Describing the problem of spatial updating with a linear SSM, we can find the optimal estimation of the desired location of the memory target by employing the Kalman filtering approach. Kalman filters provide the optimal estimation for a linear SSM, assuming that the noise follows a Gaussian distribution (Kalman, 1960). In our model described with Equations (1) and (2), the optimal estimation of the target location ${\hat{TR}}_{k}$ using Kalman filtering can be obtained with the following recursive equation:

(3) $${\hat{TR}}_{k}={\bar{TR}}_{k}+KG_{k}(EP_{k}-(TS-{\bar{TR}}_{k}))$$

where ${\bar{TR}}_{k}$ is the estimation of the target location based on the dynamics described in Equation (1) and ${\hat{TR}}_{k}$ denotes the corrected estimation using the available measurements *EP*_*k*_ (This process corresponds to the “correction" box in Figure 2A). Following Kalman filter theory, the optimal value for *KG*_*k*_ which is known as Kalman gain can be calculated iteratively with the following equation:

(4) $$KG_{k}=-\Sigma_{k}^{TR}{\left(\Sigma_{k}^{TR}+\sigma_{EP}^{2}\right)}^{-1}$$

with

(5) $$\Sigma_{k}^{TR}=(1-KG_{k\;-\;1})(\Sigma_{k\;-\;1}^{TR}+\sigma_{TR}^{2})$$

where $\Sigma_{k}^{TR}$ denotes the estimated error variance. We will use this estimation as an assessment of uncertainty for the estimation of ${\hat{TR}}_{k}$. We used this model for both saccadic and smooth pursuit eye movements. These two kinds of eye motion differ in several aspects. The first important difference as we already mentioned is the efference copy signal which drives them. The other difference is uncertainty about noisy observations, i.e., eye position. Knowing that saccades are jerky rapid eye movements, the uncertainty about eye position signal increases around the time of the saccade, whereas smooth pursuit is slow and almost accurate and therefore the uncertainty about eye position signal is much less compared to saccades. The uncertainty about eye movement is captured in the variance of additive Gaussian noise *n*_*k*_. In our simulations we considered $\sigma_{EP}^{2}>>\sigma_{TR}^{2}=0.01$ during saccades but $\sigma_{EP}^{2}\approx \sigma_{TR}^{2}=0.01$ for smooth pursuits during the eye motions.

#### 2.5.2. A non-linear state space model

In the previous section, we explained the linear SSM that describes the dynamics of spatial updating of memory visual targets in both saccadic and smooth pursuit eye movements in system level. Here, we went a step further and asked how this dynamics can be represented in the neural level. Therefore, we proposed the following non-linear SSM for the problem of spatial updating of remembered visual targets to make a bridge between the Marr's first level model to a Marr's second level model:

(6) $$TR_{k\;+\;1}=f(TR_{k},EC_{k},w)+\nu_{k}$$

where *TR*_*k*_ (as defined before) is a D-dimensional vector representing the memory target position in the retinotopic frame of reference (Here we explain the mathematics for *D* = 1; The extension to *D* = 2 is straight-forward and is presented in the Supplementary materials), again *EC*_*k*_ denotes the efference copy signal, and *f* is a non-linear function and represents the model dynamics. In our model *f* is implemented through a radial-basis-function neural network (RBFNN). More intuitively, the RBFNN represents a population of neurons with Gaussian tuning curves. In our model we are interested in the dynamics of the population of neurons that represents our state space during saccadic and smooth pursuit eye movements. As explained in the previous section, $\nu_{k}\sim N\left(0,\sigma_{TR}^{2}\right)$ shows the error in the estimation of *TR*_*k*_, the memory target position. Finally, *w* is a vector of the neural network parameters, so we refer to this vector as “the model parameters” in the rest of the article.

The architecture of our non-linear SSM is depicted in Figure 2. The main part of the model is the box including the three layer RBF neural network which represents the state space. The inputs are fed into the input neurons and directly propagates to the intermediate layer. The intermediate layer of this neural network consists of a population of RBF neurons with the following bell-shaped tuning curves (Zhang, 1996):

(7) $$a_{i}(x,t)=A_{i}\exp\{-\frac{{\left(x-\mu_{i}\right)}^{2}}{\sigma_{i}^{2}}\}+B_{i}\;for\;i=1,2,...,P$$

where *a*_*i*_ is the activity of the *i*^*th*^ neuron to an input *x* at time *t*, *P* denotes the number of neurons in the population, μ_*i*_ shows the gaze-centered preferred position of the *i*^*th*^ neuron, *A*_*i*_ and *B*_*i*_ show the peak and background firing rates, respectively, and σ_*i*_ is its corresponding tuning curve width. Figure 2B shows the tuning curves of neurons in the population. In this simple structure, the tuning curves of the RBF layer neurons can be considered equivalent to their receptive field. The output of the neural network is the predicted location of the memory target based on the population activities in the intermediate layer:

(8) $$TR_{k\;+\;1}=\sum_{i\;=\;1}^{P}w_{i}^{c}a_{i}(TR_{k}-EC_{k})+\nu_{k}$$

where $w_{i}^{c}$ represents the fixed post-connection weights. These connection weights are proportional to the center of the Gaussian tuning curve of the units (in the intermediate layer) they are starting from ($w_{i}^{c}\propto \mu_{i}$). The values assigned to these weights ($w_{i}^{c}$) assures a decoding mechanism similar to the center of mass calculation (see the Supplementary materials section). Equation (8) assumes that the estimated target position is integrated with the efference copy at the current time point and activates the neural population in a way to estimate the target position in the next time point. ν_*k*_ shows the error in this estimation.

To find the memory target position (*TR*_*k*_) and also the neural activity parameters, we again used Kalman filtering approach. EKF is a powerful and efficient extension of Kalman filter and provides approximately optimal estimates of the states for a non-linear SSM (Anderson and Moore, 2012). Our model is non-linear and its parameters (the parameters of the RBF units which determine the activity of these neurons through time) are unknown. Therefore, the model parameters ($A_{i}^{k},\sigma_{i}^{k}$)^2^ must be estimated at each time point *k* as well as the memory target position (*TR*_*k*_). As a result, using a regular Kalman filter is not applicable in our model. With this aim, we employed a more sophisticated approach known as dual estimation method (Wan and Nelson, 2001). In this approach at each time point, in one stage, the model parameters ($A_{i}^{k},\sigma_{i}^{k}$) are estimated using the input data and the states (*TR*_*k*_); then in the other stage, the states are predicted using the input data and the estimated model parameters in the previous stage. This process is demonstrated schematically in Figure 2C. This interleaved approach, alternating between two stages, is known as Expectation-Maximization in signal processing literature (Gupta and Chen, 2011). With this approach, one can estimate both the model parameters and hidden states just using the noisy sensed inputs. As mentioned before, this model makes a bridge between Marr's first level (computational level) and Marr's second level (algorithmic level). Therefore, explaining biophysical/biological mechanism for how the Kalman filter is implemented in neural circuitry is out of the scope of this paper.

In order to optimally estimate the memory target position (*TR*_*k*_) and model parameters ($A_{i}^{k},\sigma_{i}^{k}$) with Kalman filtering approach, a generative model for evolution and observations of these parameters is required. We presented the equation that describes the evolution of (*TR*_*k*_) in Equations (6) or (8). The inputs that brain receives as noisy observation is the noisy eye position signal as described in Equation (2).

We assume a simple random walk for neural activity parameters ($A_{i}^{k},\sigma_{i}^{k}$) evolution equation as

(9) $$w_{k\;+\;1}=w_{k}+r_{k}$$

where *w* = [*A*_1_, σ_1_, *A*_2_, σ_2_, …, *A*_*P*_, σ_*P*_] is defined as model parameter vector. *r*_*k*_ ~ *N*(0, Σ_*w*_) is the process noise in estimation of the model parameters. The Equation (6) is considered as observation equation for model parameters space.

Having developed the generative models for both target position space and model parameters space, one can now estimate theses variables through time optimally using the Kalman filtering approach. Given the observed eye position signal *EP*_*k*_ and the previous estimate of memory target position ${\hat{TR}}_{k-1}$, as well as the statistics of the observation noise in Equation (2), the estimation of memory target position for the next time step can be calculated using Equation (3) (Note that here ${\bar{TR}}_{k}$ is obtained based on Equations 8), (4), and

(10) $$\Sigma_{k}^{TR}=(1-KG_{k})(F_{k\;-\;1}\Sigma_{k}^{TR}F_{k\;-\;1}^{T}+\sigma_{TR}^{2})$$

where $\Sigma_{k}^{TR}$ denotes the estimated error variance. We will use this estimation as an assessment of uncertainty for the estimation of ${\hat{TR}}_{k}$. *F*_*k* − 1_ is the Taylor linear approximation of non-linear function *f* around the previous estimation, ${\hat{TR}}_{k-1}$. The detail of calculating *F*_*k* − 1_ can be found in the Supplementary materials. Finally, the current estimation of the target position, ${\hat{TR}}_{k}$ is fed back to the input neurons which propagates this vector to the RBF layer where this vector is coded as the population activity according to Equation (7).

Similar procedure is used for estimating the model parameters, *w*_*k*_, through time. Another extended Kalman filter approach is used for the estimation of the model parameters as follows

(11) $${\hat{w}}_{k\;+\;1}={\hat{w}}_{k}+KG_{k}^{w}\left({\hat{TR}}_{k\;+\;1}-f({\hat{TR}}_{k},EC_{k},{\hat{w}}_{k})\right)$$

where $KG_{k}^{w}$ is the Kalman gain for the second Kalman filter and is optimally calculated as

(12) $$KG_{k}^{w}=\Sigma_{k}^{w}C_{k}^{wT}{\left(C_{k}^{w}\Sigma_{k}^{w}C_{k}^{wT}+\sigma_{TR}^{2}\right)}^{-1}$$

with

(13) $$\Sigma_{k}^{w}=(I-KG_{k}^{w})\Sigma_{k\;-\;1}^{w}$$

in which $\Sigma_{k}^{w}$ denotes the estimated error covariance matrix for parameters *w*, and $C_{k}^{w}$ is the linear approximation of function *f* around $w={\hat{w}}_{k}$.

Here we are using the same architecture to model spatial updating across both saccadic and smooth pursuit eye movements. Our model is designed to make an online estimation of memory target position during these two kinds of eye movements. Employing the Dual EKF as described before suffers from a drawback; the dual extended Kalman filter makes an overdefinite estimation about the model parameters; therefore, one cannot see the effect of increase in uncertainty in the dynamics of the model. For this reason, we combined the approach described above with the sequential “growing and pruning” learning strategy which is a modified version of the work presented by Huang et al. (2005). The short training time and fast convergence of this method makes it an efficient approach for real time estimation of the model parameters. We know that there are a large number of visual neurons in SC (Mays and Sparks, 1980). Each is spatially selective to visual stimulus presented in a specific position relative to the gaze position. In the proposed method we only estimate the model parameters for the subset of neurons that were activated in response to the memory target. This makes the online estimation more efficient. The details of our proposed learning method are provided in the following.

In this method, we define a criterion named neuron contribution (similar to the neuron significance criterion in Huang et al., 2005) which determines the contribution of each neuron of the intermediate layer in the output of the model as

(14) $$E_{cont}(i)=\|A_{i}{\|}_{1}\int \phi_{i}(x)p(x)dx,\;for\;i=1,2,...,P$$

where $\phi_{i}\left(x\right)=\exp\left\{-\frac{{\left(x-\mu_{i}\right)}^{2}}{\sigma_{i}^{2}}\right\}$ shows the tuning curve of the *i*th neuron and *p*(*x*) is the probability density function of input signal *x*. Kalman filter approach estimates a Gaussian distribution over the hidden states which is also the feedback input in our proposed model (The dynamics of model parameters are presented in Equation 9). Therefore, at each step of dual Kalman filter the distribution *p*(*x*) is estimated by the first Kalman filter and can be used in the second Kalman filter to estimate the parameters of the model. In the pruning and growing method a neuron in the intermediate layer gets activated/deactivated based on its contribution in the model output. By activation, we mean that the neuron is allocated to the model and its corresponding parameters are estimated by the Kalman filtering approach described in Equations (11)–(13). Assume that ${\hat{TR}}_{k}$ is the *k*th input to each neuron in the intermediate layer of the network and $f\left({\hat{TR}}_{k},EC_{k},w_{k}\right)$ is the most recent output of the neural network. Therefore, the error is defined as

(15) $$e_{k}={\hat{TR}}_{k\;+\;1}-f({\hat{TR}}_{k},EC_{k},w_{k}).$$

Using the definition provided in Equation (14), the neuron contribution for the *i*^*th*^ neuron of the population with a Gaussian tuning curve with position selectivity of μ_*i*_ and width of σ_*i*_ and an input *TR*_*k*_ with a Gaussian distribution with mean ${\hat{TR}}_{k}$ and variance of $\Sigma_{k}^{TR}$ can be calculated as

(16) $$|E_{cont}(i)|=\frac{\|A_{i}\|\sigma_{i}}{\sqrt{2\Sigma_{k}^{TR}+\sigma_{i}^{2}}}\exp\{-\frac{{\left({\hat{TR}}_{k}-\mu_{i}\right)}^{2}}{2\Sigma_{k}^{TR}+\sigma_{i}^{2}}\}.$$

A neuron in the intermediate layer is activated if

(17) $$|E_{cont}(i)|>e_{min}$$

where *e*_*min*_ is the minimum expected error. Activation of the *i*_*th*_ neuron means that *A*_*i*_ = *A*_0_ and σ_*i*_ = σ_0_ (The initial values for an activated neuron). The condition in Equation (17) ensures that a neuron in the hidden layer is activated when its activation improves the learning accuracy. After the growing step, the parameters of the network are updated and moreover, the neurons in the intermediate layer are checked for possible pruning that improves the learning accuracy. In other words, all the model parameters are updated using the same process as explained above in the extended Kalman filter learning method in Equations (11)–(13) and then the neurons with a contribution of less than *e*_*min*_ are deactivated in the intermediate layer (i.e., *A*_*i*_ = 0). More details on the steps of the method can be found in Supplementary materials.

## 3. Results

In this section we tested the model described above to see if it can replicate the behavioral and neurophysiological evidence for spatial updating in the oculomotor system and if it can make further predictions about the dynamics of neural and population behavior of visual remapping. In the first section of the results, we will consider the pursuit-saccade task and show the results our model provides being trained on this task. Then, we will consider the results when our model is trained on the saccade-saccade task. As we shall show, our model accounts for different aspects of these two types of eye movements in behavioral and neural levels.

### 3.1. Spatial updating in the pursuit-saccade task

In this section, we evaluated our proposed model on the spatial updating paradigm during slow continuous eye movements through a double-step pursuit-saccade task (Figure 1B). Examples of the signals used for evaluating the model are shown in Figures 3D,E. In this case, there is no “amplitude burst” so the updating model had to rely on velocity and position signals.

#### 3.1.1. Output of the model: continuous updating

**Figure 4:**
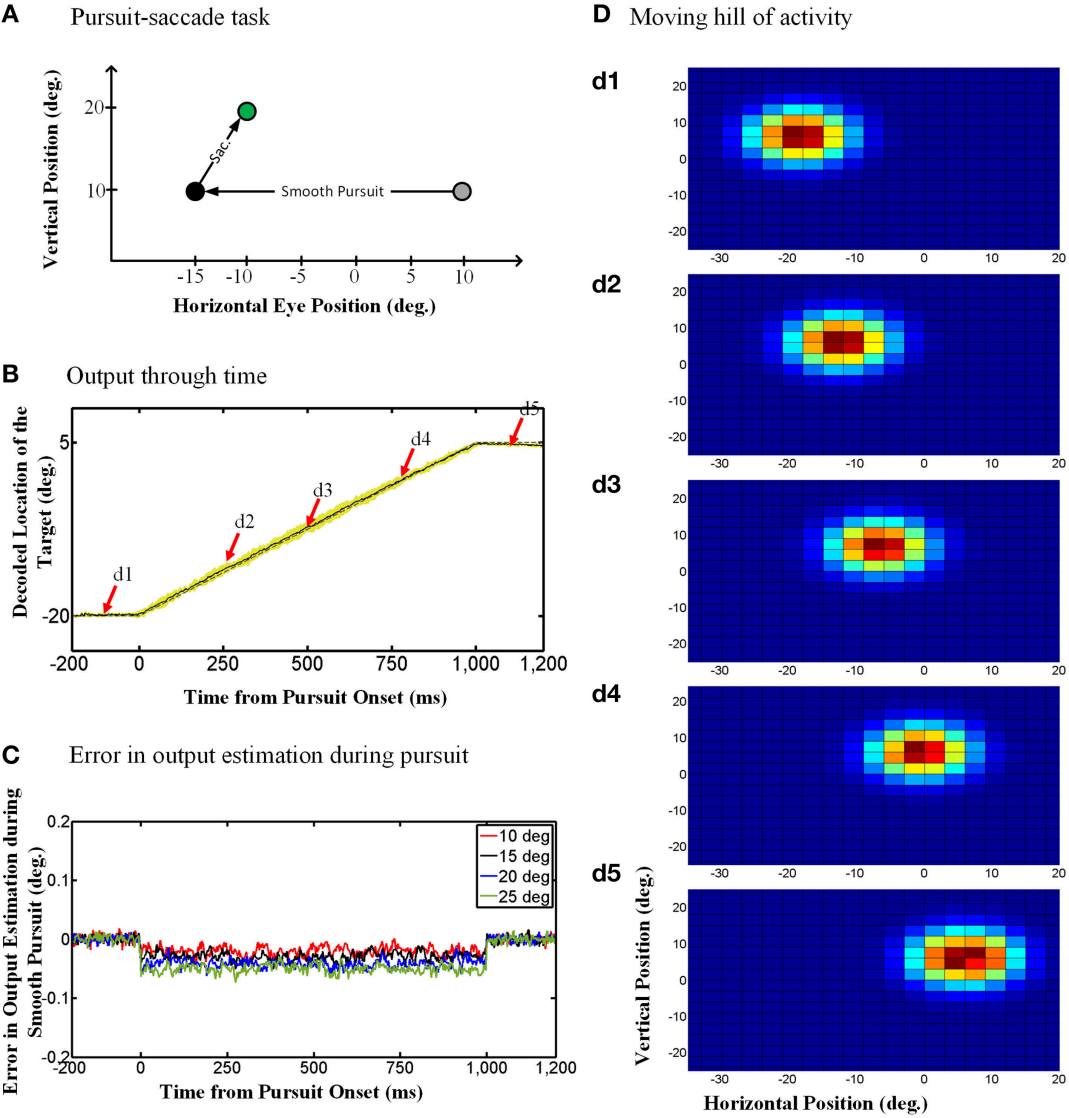
**Continuous spatial updating during smooth pursuit eye movements. (A)** Configuration of the double step pursuit-saccade task we used to evaluate our model. The initial fixation point (gray circle) is placed at (10°, 10°) and the memory saccade target (green circle) is located at (−10°, 20°) in space coordinates. The subject follows the fixation point from gray circle to the black one [at (−15°, 10°)] where he is supposed to make a saccade to the remembered target (green circle). **(B)** Model output through time. While the subject is following the fixation point from gray circle to the black one, the position of the remembered target (solid curve) is getting updated continuously through time. This diagram shows the horizontal retinotopic position of the target through time (solid curve). The yellow shadow shows the standard deviation of this output over 100 trials. **(C)** Error in the estimation of the target position during smooth pursuit eye movements (for different pursuit amplitudes). The negative values show a tracking behavior in smooth pursuit rather than a predictive one. Also the error increase with the amplitude of the smooth pursuit. **(D)** Moving hill of population activity through time. The population activities which code the retinotopic position of the saccade target is moving continuously from its initial position in **(d1)** to its final position in **(d5)**. The timing of the **(d1–d5)** are marked up in **(B)** with red arrows. The color scale indicates activity in individual units, with blue corresponding to minimal activity and dark red corresponding to maximal activity as depicted in Figure 3A.

The “behavioral” outputs of the model are presented in Figure 4. The diagram in Figure 4A depicts the pursuit-saccade task that we used to test our model, similar to that shown in Figure 1B. Figure 4B shows the model output for this task: the estimated location of the previously viewed target in gaze-centered coordinates through time. Note that the simulations were two-dimensional, but in Figure 4B vertical eye position remained stable so only the horizontal position of the target relative to current gaze position is shown. The solid black curve in Figure 4B shows the target location averaged over 100 trials of the paradigm explained in Figure 4A. The yellow shadow shows one standard deviation over these 100 trails. The dashed gray curve shows the target position relative to current gaze direction when no noise exists (ideal behavior). As one can see, the model shows a continuous gaze-centered tracking rather than predictive updating, and follows the ideal behavior very closely.

To evaluate the consistency of these results across different trials and different pursuit velocities/amplitudes, and assess the potential implications for vision, we calculated the error between the estimated output by the model and the output obtained based on the actual eye movement. To find this error, we first found the average of the estimated output over 100 trails and then calculated the difference between the outputs obtained based on the actual eye movement and the averaged estimated output by the model. Figure 4C shows this error in degrees for smooth pursuit eye movements with the same duration but different velocities. This error in output estimation for the smooth pursuit movement is constant and negative during the eye movement, meaning that the internal representation consistently lagged relative to the direction of eye movement but did not accumulate. The error increased with smooth pursuit velocity, but note that it was always very small (range [−0.1, 0.02]). This shows near-ideal tracking behavior in slow continuous eye movement. Similar results were obtained when we simulated pursuit of different amplitudes and velocities (Figure 4C). In other words, regardless of pursuit kinematics and saccade target location, the remembered location of the target was updated continuously, with a short delay relative to the actual pursuit eye movement.

#### 3.1.2. Internal states of the model: a “moving hill” of neural activity

**Figure 5:**
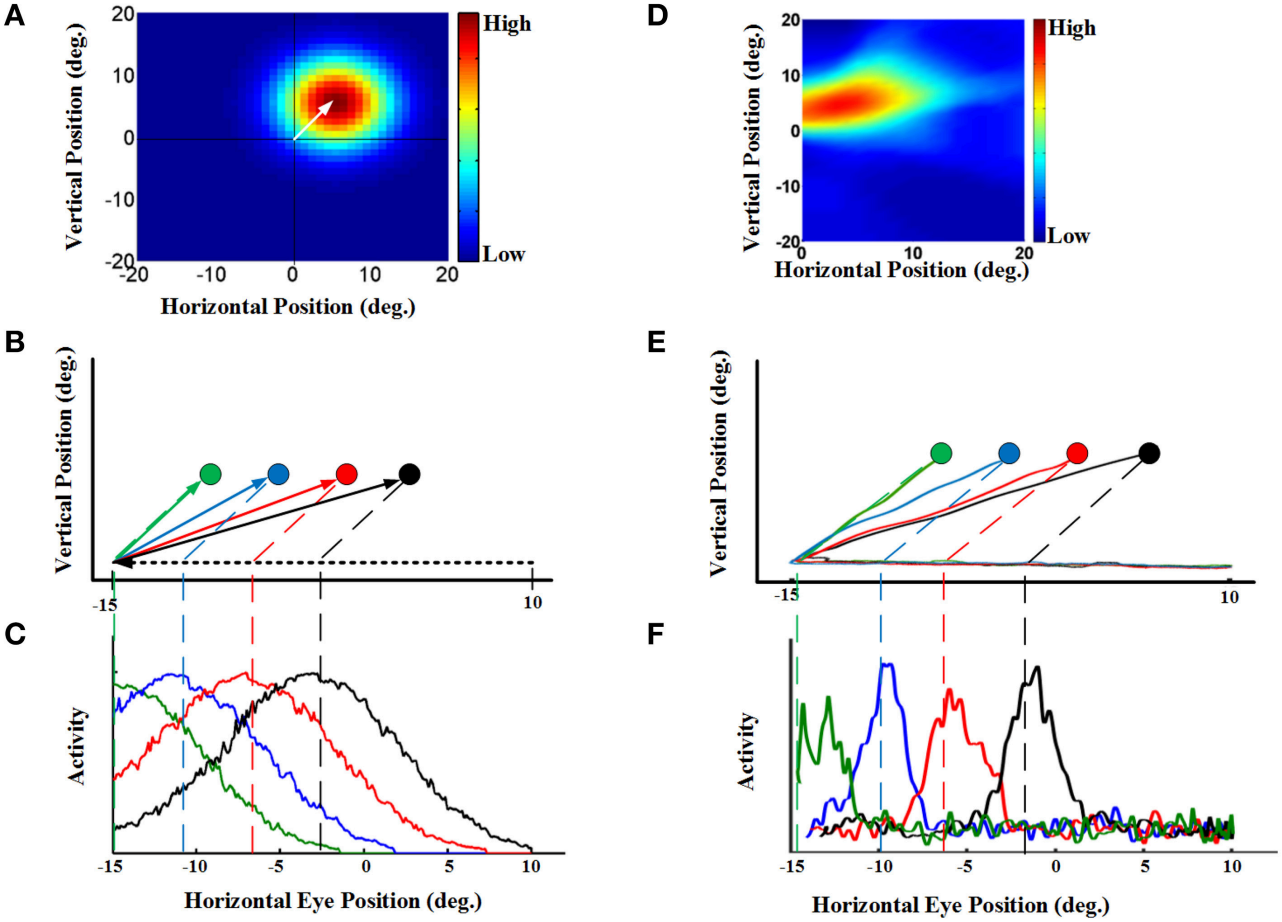
**Continuous updating of an example neuron activity during smooth pursuit eye movement [a comparison of our model to the experimental SC data in Dash et al. (2015)]. (A)** a neuron 2D receptive field centered at 5° right and 5° up relative to gaze position. **(B)** Eye movement trajectories in 2D spatial coordinates. This panel shows four trials of a 25° leftward smooth pursuit followed by a saccade to different previously shown visual targets. The circles indicate the remembered positions of the targets. The green, blue, red, and black shows the remembered positions of targets previously shown at the horizontal positions −10°, −6°, −2°, and 2°, respectively. All targets are placed at 5 degree vertical position. **(C)** This panel shows the neural activity of an example neuron with receptive field centered at 5° right and 5° up. The neural activity is plotted in terms of horizontal eye position for the trials explained in **(B)**. This neural activities for different trials are color coded. These neural activities show that the neuron responds as the remembered position of the target passes through its receptive field. **(D)** an example SC neuron with a 2D receptive field also centered at 5° right and 5° up relative to gaze position. **(E)** Actual eye movement trajectories in 2D spatial coordinates with similar configuration as **(B)**. **(F)** This panel shows the neural activity (spike density) of the example SC neuron with receptive field shown in **(D)** plotted in the same convention as **(C)**. This neural activities for different target positions are color coded. These SC neural activities are presented for comparison of the model neuron **(C)** with the SC neuron **(F)**. For complete details of the experimental methods used to obtain the data in **(D–F)** see Dash et al. (2015). Those experiments were done in accordance with Canadian Council for Animal Care guidelines and were approved by the York Animal Care Committee.

To understand how the model produced the behavior described above, we looked at both population activity (Figure 4D) and the properties of individual “neurons” in the hidden layer of the RBF neural network (Figure 5). For our single-unit analysis, we used the same conventions used by experimental neurophysiologists. Thus, Figures 5A–C (left column) provides simulations that use the same conventions utilized by Dash et al. (2015) to analyze their single-unit SC data. Corresponding plots for an actual example neuron from Dash et al. (2015), with similar properties and from a similar task are provided in the adjacent rightward column (Figures 5D–F). Figure 5A shows the eye-fixed Gaussian RF of a typical neuron, which in this case peaked 5° to the right and 5° above the fovea/gaze fixation point (similar to the example SC neuron RF shown in Figure 5D). The task details were then arranged so that remembered saccade targets would pass through the neuron's RF at different times during the pursuit eye movement. Figure 5B illustrates a series of four 25-degree leftward smooth pursuit eye movements followed by saccade to the remembered location of four different targets (Figure 5E shows similar trajectories of monkey's eye movements in (Dash et al., 2015).). Finally, Figure 5C plots the activity of our neuron as a function the same horizontal positions plotted in Figure 5B. These show that, as each target entered the neuron's RF, it became active, rising to a peak of activity when the remembered target's location crossed the peak of the RF (vertical dashed lines), and then dissipating as the eye continued to move the target out of the RF. The response was spatially selective, depending on both the RF and the target location. This pattern of simulated activity was similar for all of our hidden units and agrees exactly with actual SC visual neuron responses, like those shown in Figure 5F. The model and data also showed similar results when divided into short, medium, and long pursuit ramps (Figure S1).

Dash et al. (2015) could not directly reproduce topographic population activity, because in their experiment they varied the task for each neuron. However, they argued that by knowing single-unit properties, one can predict the population behavior (Dash et al., 2015). Specifically, the updating activity at the level of the single unit properties illustrated in Figure 5 (for both the model simulations and real data) should correspond to a moving hill of activity at the population level. Here, we demonstrate this directly by simulating SC population activity through time during the updating task in our model. This is illustrated in Figure 4D, using the same task as 4.A, and taking temporal “snapshots” at the times (d1–d5) indicated by the arrows in Figure 4B. Figure 4D shows the entire population of responses in the hidden layer of our RBF neural network. Our simulated hidden units do not have physical topography, but for illustrative purposes they have been arranged in the figure according to the peak of their RFs in a gaze-centered retinotopic map similar to that observed in the superficial layers of the SC (Walker et al., 1995; Sommer and Wurtz, 2002; Marino et al., 2008). Figure 4d1 shows the population activity 100 ms before pursuit onset. This shows a “hill-like” pattern, with the peak of activation corresponding to saccade target position relative to initial gaze, and spatially dissipating in the surrounding circle (this circle is stretched horizontally by the scale in our illustration). This pattern of population activity arises naturally from Gaussian RFs and is typical of topographically arranged visual and motor responses in the SC (Walker et al., 1995; Sommer and Wurtz, 2002; Marino et al., 2008).

More importantly, Figures 4d2–4 depict the population activity at 250, 500, and 750 ms, respectively, after the onset of the rightward pursuit movement. These panels demonstrate snapshots through time of a continuously “moving hill” of activity during smooth pursuit eye movement. This hill moves from right to left in opposition to the direction of eye motion, maintaining the location of the saccade target in gaze-centered coordinates. As a result, whenever pursuit stops (Figure 4d5) the necessary population activity was retained for transformation into a saccade. In other words, the neural population code for the remembered saccade target was updated continuously in gaze-centered coordinates during a smooth pursuit eye movement, always ready for behavior.

As argued by Dash et al. (2015) this is simply the necessary population corollary of the single unit properties reported above: the peak of the moving hill corresponds to the target entering the peak of the eye-fixed RF of some cell at one point in time (vertical dashed lines in Figures 5B,C) whereas the surrounding cells (in our virtual topographic map) show decrementing activity as a function of the distance of the remembered target from the peak of their RFs. It is this population response, combined with the output decoding process incorporated into our model, that results in the updating behavior shown in Figure 4B. In physiological terms, this decoding process could be implemented by transfer of this visual memory activity into saccade motor activity (Also observed by Dash et al., 2015).

The preceding phenomenology is similar to what neurophysiologists have reported, but here we designed the model so we know what it does and we know how it works. Thus, in our model we know the purpose of the continuous updating responses in our hidden units is to update the memory of the saccade target, and that it is causally related to this function. We know that this works through the continuous transfer of activity across the virtual retinotopic map of our hidden layer.

### 3.2. Spatial updating in the saccade-saccade task

We also used our model to simulating saccade-saccade task. To this end, we used the efference copy signal presented in Figure 3B and the eye position signal depicted in Figure 3C as the inputs of the model. As we explained in Section 2.3, during a saccade, the eye velocity or position is less reliable comparing to a smooth pursuit task and the intended eye displacement becomes a more dominant factor in updating during a saccadic eye movement.

#### 3.2.1. Output of the model: predictive updating and peri-saccadic errors

**Figure 6:**
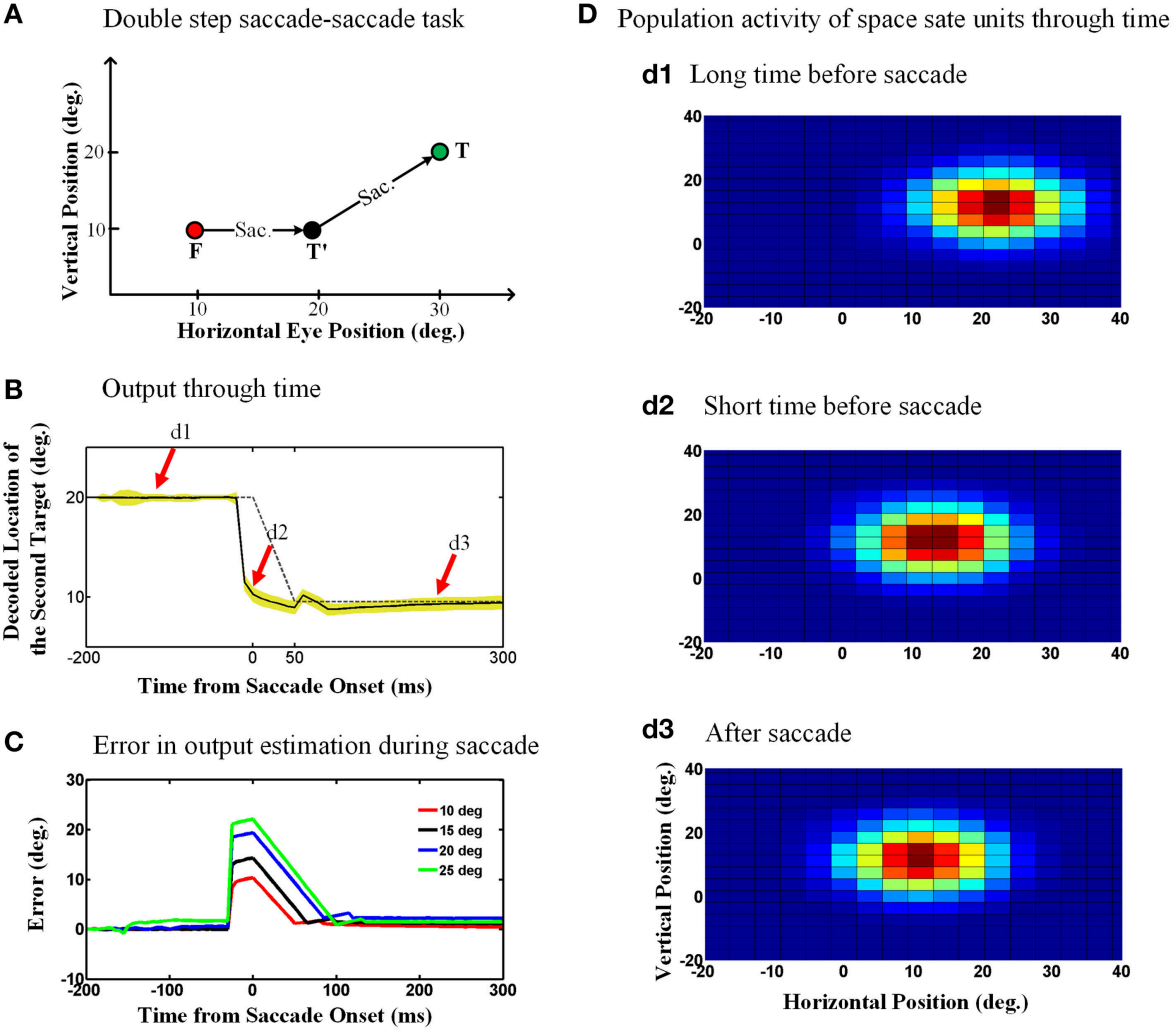
**Predictive remapping of remembered second target position during saccades. (A)** Configuration of the double step saccade-saccade task we used to evaluate our model. The initial fixation point is placed at (10°, 10°), and the first and second saccade targets are located at (20°, 10°) and (30°, 20°) in space coordinates, respectively. The subject makes the first saccade from the fixation point (red circle) to the first target (black circle) where he is supposed to make the second saccade to the remembered second target (green circle). **(B)** Model output through time. When the subject makes the first saccade from the fixation point (red circle) to the first target (black circle), the retinotopic position of second target is updated discretely and predictively. The solid curve shows the retinotopic horizontal position of the second target through time. As can be seen, this estimated position jumps predictively to its future position even before the saccade onset at time zero. The yellow shadow shows the standard deviation of the output over 100 trials. **(C)** Error in the estimation of the second target position during saccadic eye movements (for different saccade amplitudes). These positive values show a predictive behavior. Also as the saccade amplitude increases, the peak of the corresponding curve increases proportionally. **(D)** Population activity of the state space units through time. The population activity of neurons which code the retinotopic position of the second saccade target are depicted in **(d1–d3)**, respectively, at a long time before the saccade, a short time before the saccade and long after the saccade. As can be seen, the population activity jumps to its future position at a short time before the upcoming first saccade, i.e., it shows predictive remapping.

Figure 6A shows the double step saccade-saccade paradigm we used to test our model. As can be seen in this figure, the initial fixation point is located at the coordinates (10°, 10°), the first target is placed at the position (20°, 10°) and finally the second target position relative to space is (30°, 20°). Therefore, the second target position in gaze centered coordinates at the initial fixation point is (20°, 10°) and after the first saccade, the second target position relative to gaze will be (10°, 10°). Figure 6B depicts the model output which is the estimated position of the second target relative to gaze through time, 200 ms before saccade onset to 250 ms after the saccade completion. The duration of this 10-degree saccade is considered to be 50 ms. The solid curve is the output average over 100 trials with the configuration as explained in Figure 6A and the yellow shadow is the standard deviation of the predicted output over these 100 trails. The dashed curve shows the second target position relative to gaze position in an ideal case without noise. As can be seen in Figure 6B, the estimated second target position by the model shows a predictive behavior. In other words, the output (solid line) jumps predictively to its future position before the saccade onset (dashed line; actual eye movement). This is because the efferent saccade displacement signal, largely responsible for the updating, arises before the actual saccade (Figure 3).

One consequence of the predictive updating response is that there is a transient peri-saccadic mis-match between actual and remembered target position. To quantify this, we again calculated the error between the estimated output by the model and the output obtained based on the actual eye movement. Figure 6C shows the calculated error in estimation of the second target location through time for different saccade sizes. As can be seen in Figure 6C, this error arises rapidly just before the saccade, peaks around the time of saccade onset, and then drops back to zero (with some minor oscillations) after the saccade. Further, the direction of the error is always positive (in the direction of the saccade) and peaks at an amplitude approximately equal to the corresponding saccade amplitude. In our model, these observations result from the predictive behavior in the estimation of the second target position using the first saccade displacement command, followed by the influence of the eye position signal, which has a slower time course. If this influenced perception, it would predict large errors in stimulus localization just before saccades and smaller errors just afterwards (This will be discussed further in Section 4.2).

#### 3.2.2. Internal states of the model: predictive remapping in neurons and populations

**Figure 7:**
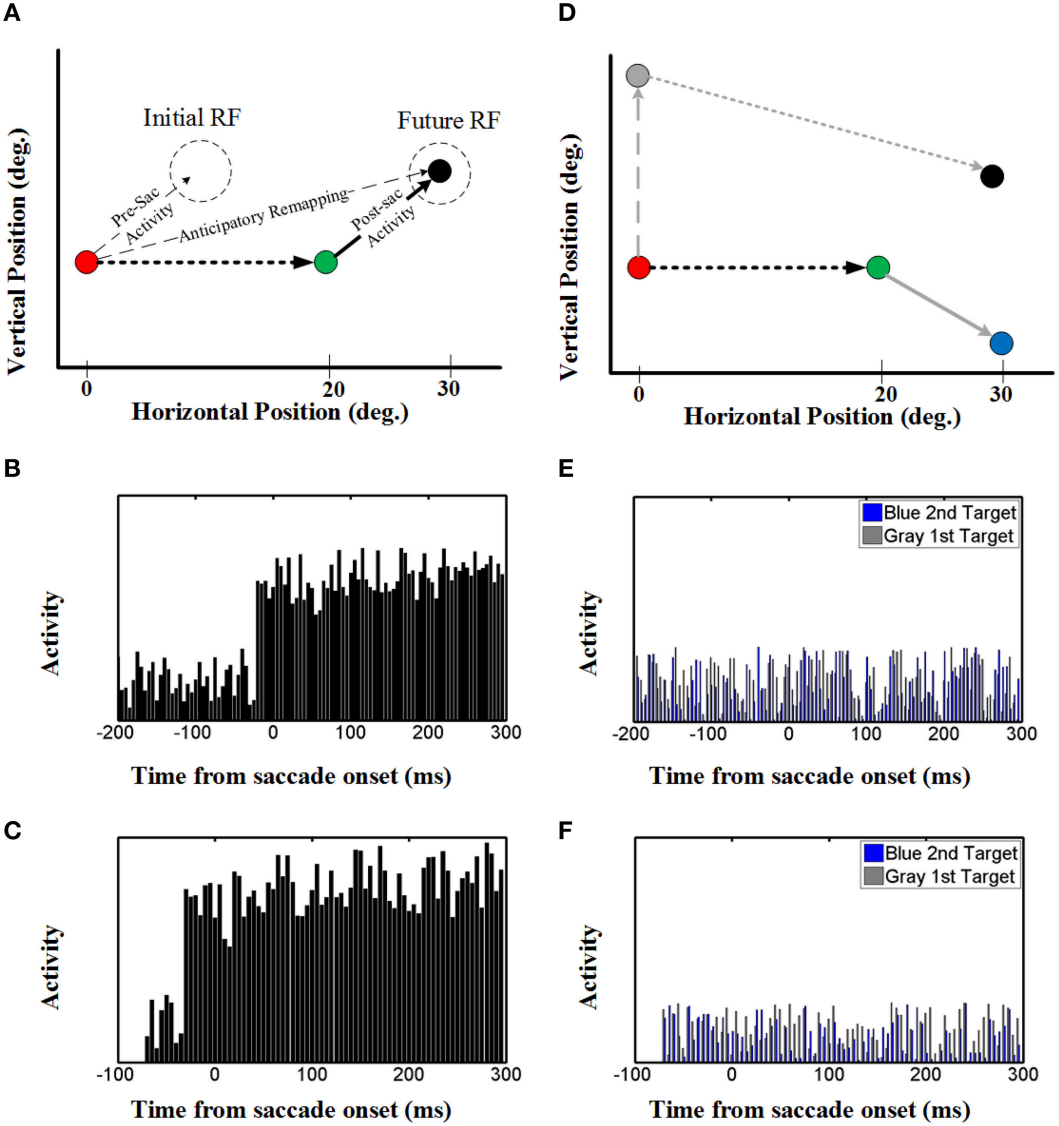
**Effect of upcoming saccade on an example neuron activity. (A)** This panel shows the double step saccade-saccade paradigm we used to find neural activity. The red, green, and black circles indicate the fixation point, intervening saccade target, and the memory target, respectively. The memory target is placed in (30, 5)° relative to the first fixation point and after a 20° rightward intervening saccade, the position of this memory target will be at (10, 5)° relative to the new gaze position. **(B)** This diagram shows the neural activity of a neuron with receptive field centered at (10, 5) relative to gaze position. As can be seen in this diagram, the neural activity (black spikes) raised before saccade onset. This shows a predictive remapping behavior of this neuron. **(C)** This diagram shows the neural activity of a neuron with receptive field centered at (10, 5) relative to gaze position in the case the second target presented close to the saccade onset (similar to Duhamel et al., 1992) experiment. As can be seen in this diagram, similar to **(B)** the neural activity (black spikes) raised before saccade onset. This shows a predictive remapping behavior of this neuron. **(D)** As control conditions, the activity of the same neuron but for a different memory target at position (30, −5) (blue circle) and the activity of this neuron for a different intervening saccade target (gray circle) are also plotted (gray spikes). **(E,F)** In the control test, the activity of this neuron does not show any specific behavior. This is due to the fact that the blue memory target is out of its receptive field before, during and after the saccade.

Once again, to understand how the decoded output of the model arose, we investigated both the population behavior and individual neurons in the hidden layer, again using techniques described by neurophysiologists. This simulation illustrated in Figure 7A replicates the conditions used in the classic Duhamel et al. (1992) experiment, with two visual stimuli presented before the first saccade (to the green target) except that we have added a second saccade to the black target to clearly define the behavioral relevance (Figure 1A). These neurons again have eye-fixed Gaussian RFs, and the geometry of the simulation has been arranged so that the RF of the neuron (initial RF) is initially far from any stimulus, whereas the first saccade brings it right over the black stimulus (future RF).

The diagram in Figure 7B shows the neural activity of the neuron mentioned above, using our standard 200 ms memory interval before saccade onset (Figures 1A,B). Initially, this “neuron” only showed random background activity because there was nothing in its initial RF. As can be seen in this diagram, the neural activity then rose abruptly before saccade onset. This shows a predictive remapping behavior of this neuron, corresponding to the dashed line labeled “anticipatory remapping” in Figure 7A. Recording from this neuron, it would look like its RF transiently shifted (remapped) toward the future RF. This neuron then shows sustained activity after the first saccade, corresponding to the location of black target (now extinguished) in its normal eye-fixed RF. Thus, at the population level (Figure 6) one can see that neural activity is predictively transferred between different neurons (i.e., from the population activated at the original eye position to the appropriate population for the final eye position), whereas at the single unit level (Figure 7A), this same phenomenon manifests itself as a predictive remapping response followed by sustained activity in cells with RFs corresponding to the final target/eye position.

These observations are very similar to the data reported by Duhamel et al. (1992) in a single step task (no saccade to the second target), except that in those experiments there was no memory lag between the visual stimulus and the first saccade. To show that this delay makes no differences in our model, we reduced the memory interval to 70 ms before saccade onset, so that there was only one time step between the arrival of the visual stimulus (Figure 3A) and the pre-saccadic efference copy (Figure 3B). (We could not reduce this further because our model structure does not allow simultaneous presentation of visual and motor inputs.) The results (Figure 7C) show that, other than the arrival of visual activation closer to the saccade, this simulation gave the same results as Figure 7B. Thus, this predictive and sustained activity of our model is in agreement with neurophysiological evidence found by Duhamel et al. (1992), and in subsequent remapping experiments (Walker et al., 1995; Umeno and Goldberg, 1997). Moreover, when these simulations are repeated over multiple trials (similar to multiple neurons) the uncorrelated noise cancels, providing a very clean updating response (Figure S2).

We also tested our neurons in two control conditions (Figure 7D) with either 1) the same final target but a different first saccade target (gray: that does not bring the final target into the neuron RF), or the same first saccade and different final target (blue, outside of the neuron RF after the first saccade). In either case, this did not result in any change in baseline neural activity (blue and gray activity plots in Figures 7E,F). As shown in Figures 7E,F, this observation was also not influenced by memory delay in our model. This shows that the predictive and sustained remapping response in our model was spatially selective, depending on the first saccade bringing the second saccade target into the neurons RF.

As a result of these single unit properties, one can again understand and predict what will happen at the population level. The population activities are shown in Figure 6D, using the same plotting conventions described above for Figure 4D. The three panels 6.d1-d3 correspond to the task shown in Figure 6A, taken at the times indicated by the red arrows in Figure 6B. Figure 6d1 again shows the typical “hill” of population activity before saccade onset. Figure 6d2 depicts population activity at a short time before saccade onset, showing a predictive jump to its final position before eye movement. Finally, Figure 6d3 demonstrates the population activity at a long time after the saccade completion. Thus, the peak of the population activity “skips ahead” in the direction of the saccade, and then is sustained after the saccade, giving rise to the decoded behavioral outputs illustrated in Figure 6B.

Again, we know the reason and purpose for this behavior in our model: it is to update remembered activity relevant for future behavior (here the second saccade), and the anticipatory aspect simply results from the timing of internal saccade motor signals relative to actual delays in the eye movements.

### 3.3. Position uncertainty and expansion of receptive fields during saccades

Until now we have assumed that the input signals to our model are equally stable during fixations and saccades, but this is unlikely to be true. Saccades are very rapid and accompanied by large transient signals that are thought to add noise and uncertainty to the visual system (Harris and Wolpert, 1998; Niemeier et al., 2003; Prime et al., 2007). For example, around the time of saccades eye position signals are probably unstable, inaccurate, and possibly out of synch with real time. Speaking statistically, the uncertainty in the probability distribution of the eye position signal increases around the time of the saccade compared to the fixation conditions. This was a motivation for us to investigate that how this increased uncertainty might influence spatial updating. To investigate this we employed this input uncertainty in the learning procedure of the model (details and mathematical aspects are provided in the Supplementary Materials).

**Figure 8:**
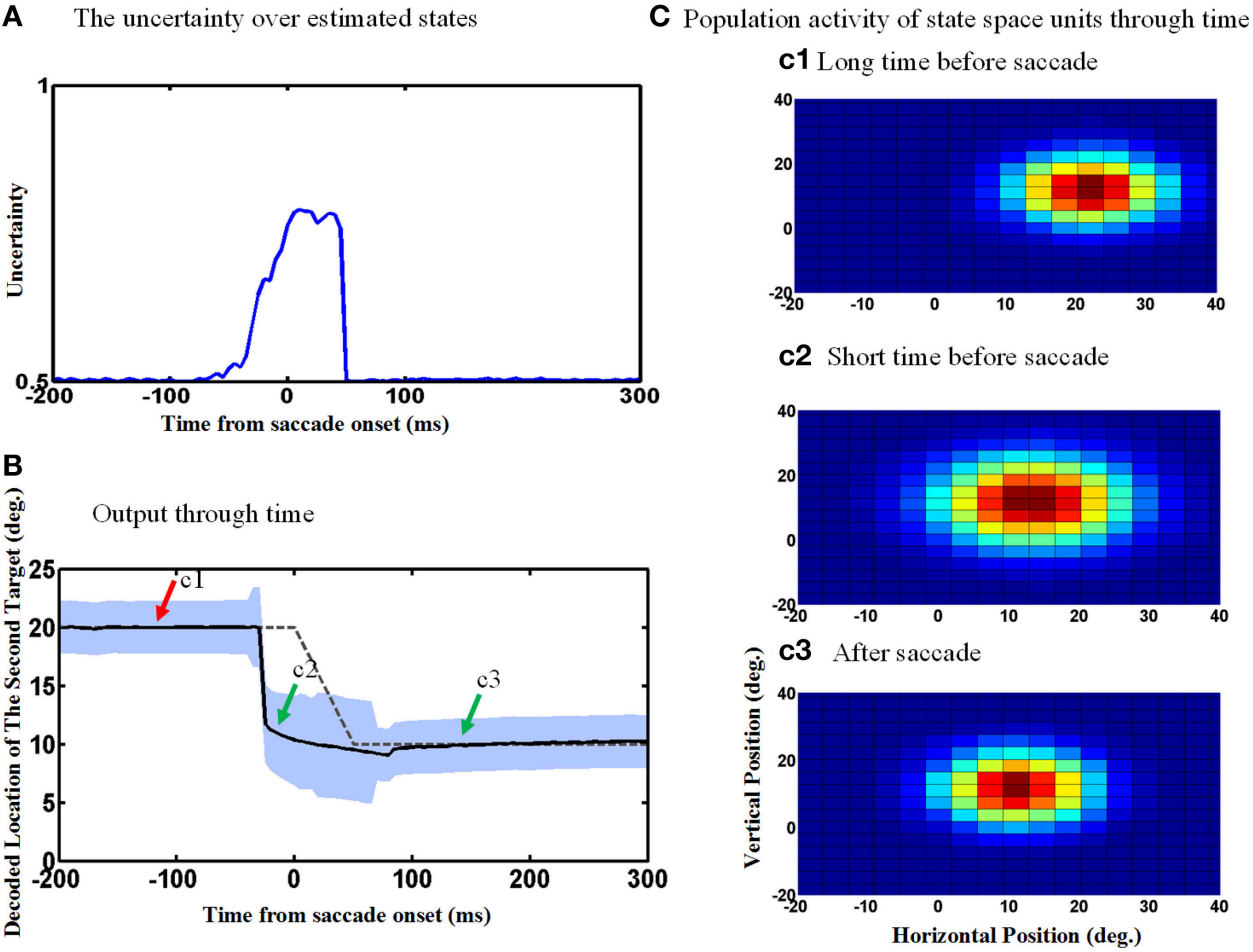
**Population activities expand during saccades due to the increase in eye position measurements uncertainty. (A)** The uncertainty over estimated states by Kalman filter. The uncertainty is measured by the width of Gaussian distribution. **(B)** Model output through time. The estimated second target position jumps predictively to its future value even before the upcoming eye movement. Moreover, the width of the population activities increases before the upcoming saccade and this broadening continues during the saccade as shown by the blue shadow. As depicted, more neurons get activated when the width of distribution (shown in **A**) increases. **(C)** Population activity of the state space units through time. The population activity of neurons which code the retinotopic position of the second saccade target are depicted in **(c1–c3)**, respectively, at a long time before the saccade, a short time before the saccade and long after the saccade. As can be seen, the population activity jumps to its future position and also expands at a short time before the upcoming first saccade.

Figure 8 repeats the simulations shown in Figure 6, except now allowing for the uncertainty of eye position signal around the time of the saccade which results in an increase in the uncertainty of the estimated internal states around the time of the saccade. This internal states uncertainty (shown as the width of the input Gaussian distribution of the estimated hidden states) is depicted through the time course of the spatial updating task in Figure 8A. Simulating the double-step saccade-saccade task under this new condition produced model output (Figure 8B) that still showed the anticipatory “jump” (solid black line relative to the dashed line for the actual saccade). Likewise, the population activity of hidden-layer neurons (Figure 8C) again jumps, but also expands a short time before the saccade (Figure 8c2). The time course of this expansion is represented by the vertical width of the blue shaded area in Figure 8B. The expansion begins at the onset of the predictive jump before the saccade (dashed line), and occurs continuously until just after the saccade, after which the population activity returns to its original size (Figure 8B/mark c3 and Figure 8c3).

**Figure 9:**
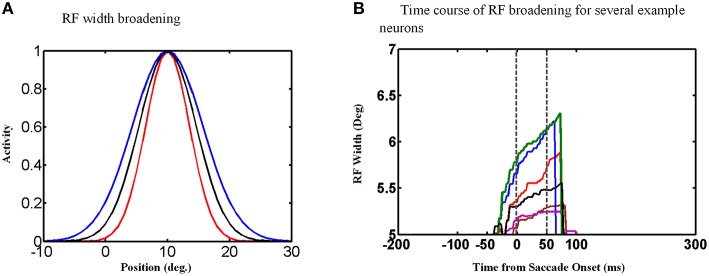
**RF width of state space units through time. (A)** Shows how the RF of an example neuron broadens during the saccade. **(B)** This figure shows the RF widths of several neurons which the remembered target falls in their RFs through time for a 10° saccade. As can be seen in this figure, the RF size starts to increase before saccade onset and returns back to its actual size shortly after the saccade completes.

An examination of the behavior of single units explains the behavior of the population and its output. The width of the Gaussian RFs increases around the time of the saccade (Figure 9A). In this figure, red curve shows the RF of an example neuron long before the saccade, black curve shows this neuron RF shortly before the saccade and blue curve shows its RF during the saccade. Figure 9B shows the time course of some example neurons which the memory target falls in their RF. As can be seen the width of RFs start to increase before saccade onset and broaden continuously during the saccade, and finally return abruptly to their normal fixation size shortly after the saccade. As a result of this transient RF broadening, a wider swath of neurons are activated during the updating task (Figure 8c2). This leads to two predictions: expanding RFs, and broader recruitment of neurons around the time of a saccade.

## 4. Discussion

The primary aim of this study was to determine if an artificial network developed to perform a simple double-step spatial updating task could also replicate the major neurophysiological phenomena that have been associated with spatial updating and remapping during saccades and pursuit eye movements. To do this, we developed a state-space model (SSM) to study the dynamics of spatial updating across eye movements. The proposed model follows a dual EKF structure which is well-developed to study both behavioral and neural population activities through time. Further, depending on the inputs to this model, it is able to switch between simulating updating saccade targets across either smooth pursuit or an intervening saccade, much like the actual brain. During smooth pursuit movements, the proposed model demonstrated a continuously moving hill of activity related to the remembered target for a subsequent saccade, replicating the results of Dash et al. (2015). Moreover, during saccades our model showed a predictive “jump” in memory-related population activity to its future position, replicating the results of several “predictive remapping” studies (Duhamel et al., 1992; Walker et al., 1995; Umeno and Goldberg, 1997). Thus, the same general model structure was able to account for spatial updating / remapping during both saccadic and smooth pursuit eye movements, by using different input signals. Furthermore, the proposed model provides a new prediction that population activities expand during the saccadic eye movement. To our knowledge, no other model has been able to explain so many phenomena associated with updating/remapping, based on the single unifying concept of using corollary discharge to retain visual information in a gaze-centered frame.

### 4.1. Neurophysiological confirmation/predictions of the model

In studies of spatial updating that looked at motor activity in the double-step task, it is clear that plans for saccades are updated to compensate for intervening eye movements in a spatially specific fashion (e.g., Sparks and Mays, 1983; Medendorp et al., 2003). It is less clear how motor updating relates to the updating of visual signals during saccades (Duhamel et al., 1992; Zirnsak et al., 2014). However, it is noteworthy that most of the brain areas which show alterations in visual RFs around the time of saccades also show signals related to saccades (Duhamel et al., 1992; Walker et al., 1995; Umeno and Goldberg, 1997; Dash et al., 2015). Therefore, it may be best to consider an integrated visual-motor approach to this question.

One structure that has been implicated in both saccade production and spatial updating is the SC (Walker et al., 1995; Sommer and Wurtz, 2000; Dash et al., 2015). Dash et al. recently employed a double step pursuit-saccade task to study updating of a memory visual target during smooth pursuit eye movements in the SC. The authors reported that almost all of visual neurons in their dataset showed continuous updating during smooth pursuit, whereas motor saccade signals were only updated at the time of the saccade (Dash et al., 2015). Our model, trained on a similar task, showed similar results in single neuron level as in the visual activity reported by Dash et al. (2015). Moreover, in our model we could directly observe that the corresponding population of SC neurons shows a continuously moving hill of activity, as predicted by Dash et al. (2015) from their data. Furthermore, our model showed that this continuous updating lags the actual eye movement, a prediction that has not yet been tested. As proposed by Dash et al. (2015), it is essentially certain that our model would produce similar predictions for updating across other types of slow eye movement, for example head motion through space, if this were included in our simulations (Medendorp et al., 2003; Klier and Angelaki, 2008).

The influence of saccades on visual signals has mainly been studied in single-step saccade experiments where animals were not explicitly required to retain memory of the additional visual target for any behavioral purpose. In 1992, Duhamel and colleagues showed that LIP neurons can respond to a visual stimulus in their future RF after a saccade even before the eyes move (Duhamel et al., 1992). In other words, they found that parietal RFs are remapped to their future position predictively in order to compensate for the upcoming eye movement. After that, similar remapping evidence showing similar compensatory predictive mechanism have been found in the FEF (Umeno and Goldberg, 1997) and the SC (Walker et al., 1995). Our model produced similar remapping-like results in single neuron level (Figure 7), whereas at the population level, our model demonstrated a jump in hill of activities predictively to its future position (Figure 6). One can thus see how remapping and updating could appear to be two different things when viewed from these two different perspectives, but in our model these were simply two different ways of describing the same simulation. Further, in our model, this did not depend on timing of the stimulus: the same results occurred whether the saccade occurred immediately after the visual stimulus (as in the single-step experiments described above), or after a more prolonged delay. Finally, in our model –trained on the double step task– we know *a priori* the purpose of these phenomena: to place neural activity at the appropriate location to be ready for a second saccade from the future eye position.

The logical implication of these results is that there may also be no fundamental difference between the mechanisms for updating gaze-centered of visual memory responses vs. remapping visual responses in the real brain. One reason we used the double step task in our network was to compare the behavioral output of the network against ideal behavior to train the network. Likewise, before even entering a laboratory, a monkey (or human) has already undergone years of natural training on sensorimotor tasks that involve spatial integration across eye movements. Even in a single-step task where the remapped stimulus is irrelevant to the reward, it seems unlikely that the visual system could entirely ignore a highly salient stimulus flashed in the dark or dis-engage circuits that have been established through years of training. Indeed, the fact that some visual information is retained and updated relative to gaze direction after saccades in such tasks (Duhamel et al., 1992) suggests that this does not happen. Thus, visual remapping experiments may be tapping into part of a mechanism that was trained by behaviors resembling the double step task. This could be further tested by directly comparing the results of single-step and double-step tasks in areas associated with remapping. Based on our results here, we expect that anticipatory remapping and post-saccadicc retention would correlate (if they are part of the same mechanism), and that both would be enhanced when the visual stimulus becomes more relevant to the task. These factors suggest that a more unified approach, like ours, is required to understand the spatial updating of visual memory for action and visual remapping, which to date has mainly been interpreted in light of perceptual constancy (Wurtz, 2008; Higgins and Rayner, 2015). In short, we suggest that visual remapping and motor updating are part of a common visuomotor mechanism, and that perceptual constancy at the subjective level likely is influenced by training the brain on motor tasks.

Recently Zirnsak et al. tested a wider range of spatial combinations of visual stimuli and saccade metrics than those used in the original single-step remapping experiments (Zirnsak et al., 2014). Although some responses were consistent with remapping, many responses were more consistent with a shift of attention toward eye movement targets, and might explain why perceived visual space is transiently compressed toward these targets. We did not observe this shift of RFs toward the target in our model, but again it is important to note the differences between general context and task between our double-step study and both the Zirnsak et al. (2014) and classic remapping studies. First, in our model spatial updating was the only constraint, whereas the real system has other constraints such as attention and saccade production. Second, as pointed out above, it is difficult to interpret responses to task-irrelevant stimuli. These responses may have been influenced by subtle differences between labs that might influence implicit training and attention to the stimuli (as well as selection criteria for different cell types). We did not attempt to model these things here. Again, the best way to resolve these differences might be to repeat the Zirnsak recording paradigm on animals trained on a double-step task, where the task-relevance of the stimulus is known, and then compare cell responses during single-step and double-step behavior.

Our model predicts an expansion in the RFs sizes shortly before and during the saccadic eye movements. This new prediction could again be tested by mapping RFs during a double-step saccade task. In our model, this expansion in RFs is a consequence of the increase in the uncertainty of eye position signals around the time of the saccade (Hershberger, 1987; Honda, 1991; Dassonville et al., 1992; Jordan and Hershberger, 1994; Dassonville et al., 1995; Schlag and Schlag-Rey, 2002; Morris et al., 2012). In contrast, there was no such uncertainty in eye position signals in our pursuit-saccade simulations, and thus we did not observe expanding receptive fields in that task (although this physiological prediction has not been explicitly tested either). Thus, our model is able to implement different levels of uncertainty at the physiological level.

Finally, the preceding discussion implicitly assumes that there is a direct causal link between updated visual memory responses and motor responses. As in our model, the real brain would require a “decoder” to convert the visual memory activity into motor activity, i.e., for the second saccade step. This was demonstrated in Dash et al. (2015) experiment (Supplementary Figures) for pursuit-saccade task, and in experiments recording both visual and motor cells in saccade-saccade task (Mays and Sparks, 1980; Sparks and Porter, 1983; Goldberg and Bruce, 1990). The population response, combined with the output decoding process incorporated into our model results in the updating behavior shown in Figure 4B. In physiological terms, this decoding process could be implemented by transfer of this visual memory activity into saccade motor activity (also observed by Dash et al., 2015).

### 4.2. Behavioral/psychophysical confirmation/predictions of the model

Spatial localization is one of the important aspects of perception and action, and clearly depends on the integration of vision with eye movement signals. Several studies have shown that during smooth pursuit, subjects make systematic errors in localization of a briefly flashed target (Mitrani and Dimitrov, 1982; Matsumiya and Uchikawa, 2000; Brenner et al., 2001; Van Beers et al., 2001; Rotman et al., 2004; Kerzel et al., 2006; Blanke et al., 2010). Humans tend to mislocalize the flashed stimulus in the direction of pursuit (if the stimulus presented along the pursuit direction) and away from the fovea (if stimulus presented orthogonal to the eye movement trajectory Kerzel et al., 2006).

Likewise, many studies have reported peri-saccadic mislocalization of visual stimuli, starting before the saccade, peaking at saccade onset, and ending just after the saccade (Honda, 1989, 1991, 1993; Dassonville et al., 1992; Morrone et al., 1997; Lappe et al., 2000; Georg et al., 2008). This is completely in agreement with the error graph calculated on our model output shown in Figure 6C. Therefore, the big error around the time of the saccade is consistent with psychophysical evidence showing that humans most likely mislocalize visual targets flashed around the time of the saccade. In other words, our model is consistent with previous accounts that link peri-saccadic mislocalization or saccadic suppression of displacement to the mechanism of spatial updating (Awater and Lappe, 2006; Melcher and Colby, 2008; Van Wetter and Van Opstal, 2008; Hamker et al., 2011; Ziesche and Hamker, 2011). One potentially useful aspect of our model is that it could link mechanistic (remapping) and computational (Niemeier et al., 2003) approaches to understanding peri-saccadic perceptual errors, because it is able to implement uncertainty at the physiological level. However, this goes beyond the scope of the current paper.

### 4.3. Comparison to previous neural-net style updating/remapping models

In recent years, several computational models have been proposed to address various questions related to behavioral and neural aspects of peri- and trans-saccadic phenomena of visual perception (Zipser and Andersen, 1988; Quaia et al., 1998; Xing and Andersen, 2000; Niemeier et al., 2003; White and Snyder, 2004; Hamker et al., 2008a,b; Keith et al., 2010). These models can be categorized based on the extraretinal signals which are used and the way these signals are combined with visual signals (Hamker et al., 2011). Some of these models use a continuous eye position signal which starts before the actual eye movement and remains incomplete for a short time after the eyes land in their final position (White and Snyder, 2004). An eye position signal with these characteristics has been recently found in LIP, MT, MST and VIP (Morris et al., 2012). It has been suggested that such signals would become uncertain around the time of a saccade (Hamker et al., 2011) but this was not incorporated into previous models. We used an eye position signal based on Morris et al. (2012) in our proposed model but we also incorporated the increase in uncertainty of eye position signal around the time of the saccade. In this way our model could make new prediction about the dynamics of RFs modulation around the time of the saccade, i.e., that RFs are broadened.

The other category employed an intended eye displacement (and not eye position) as efference copy signal for remapping visual information (Quaia et al., 1998; Keith et al., 2010) or for improving the visual processing around the saccade target via increasing the capacity of visual processing by changing the structure of RFs (Hamker et al., 2008a). Keith et al. developed a three layer neural network to study the dynamics of spatial updating. They used three different signals, a transient visual response to the first saccade target, a motor burst signal which starts before the saccade or an eye-velocity signal, as efference copy to study the effects of these signals on spatial updating. They found moving population activity for eye-velocity efference copy, stretching population activity to the final position for motor burst signal and jumping when transient visual signal was used. We used motor-burst like efference copy which encodes intended eye displacement as efference copy for saccadic eye movement. This resulted anticipatory receptive modulation during saccadic eye movements in our model. Finally, eye velocity has been used to model continuous updating of behavior in some “black box” models (Medendorp et al., 2002, 2003). To our knowledge, the current study is the first (other than conference abstracts; SFN; Keith et al., 2006; VSS; Mohsenzadeh and Crawford, 2015) to use eye velocity as an efference copy for smooth pursuit eye movement to model continuously updating RFs.

### 4.4. Broader implications

Our study demonstrates that it is possible to demonstrate how perceptual, behavioral, computational, and physiological aspects of a problem here spatial updating and remapping of visual signals can be successfully integrated. More specifically, we show that many of the observations that have been made related to remapping or updating of visual signals can be explained in terms of the motor aspects of the system they serve, in this case the generation of a future saccade based on retained and updated visual information. This suggests that visuomotor control influences perception, but ultimately these systems share common goals (what is it, where is it, and what can it do?). Thus, this view does not discount the importance of perception and perceptual learning. For example, we have recently found evidence that pursuit updating signals are also active in the presence of vision (Dash et al., 2016). In this case, mismatches between updated and perceived locations would be subjectively disconcerting and disrupt trans-saccadic perception (Melcher and Colby, 2008; Prime et al., 2011). Egocentric updating also needs to interact with allocentric cues in normal environments (Byrne et al., 2010). Thus, visual coherence and interactive visuomotor behavior could cooperate to calibrate this system. Consistent with this, many studies have shown that perceptual adaptation follows motor adaptation (Rosenkranz and Rothwell, 2012; Wong et al., 2012; Darainy et al., 2013). In simple terms, insomuch as the world is stable, and the brain is able to correctly represent the world for accurate behavior, then we should also perceive the world as stable.

## Author contributions

YM and JDC designed the research. YM designed and implemented the model. YM and JDC analyzed and interpreted the results and wrote the manuscript. SD tested the model by analyzing and providing data from Dash et al. (2015).

### Conflict of interest statement

The authors declare that the research was conducted in the absence of any commercial or financial relationships that could be construed as a potential conflict of interest. The reviewer WE and handling Editor declared their shared affiliation, and the handling Editor states that the process nevertheless met the standards of a fair and objective review.

## Footnotes

^1^As in our model the time step is constant Δ*t* = 2*ms*, eye velocity sequence is multiplied by this constant to make its unit commensurate with eye direction in the case of saccade. In this way the model uses the same set of parameters.

^2^For simplicity, we assume *B*_*i*_ = 0 and the baseline can be captured in the additive noise ν_*k*_.

## Supplementary material

Figure S1 **Spatial updating behavior for different smooth pursuit lengths/durations. (A)** Eye movement trajectories in 2D spatial coordinates. This panel shows four leftward constant velocity smooth pursuit ramps (18°, 22°, 26°, and 30°) followed by a saccade to different previously shown visual targets. The green, blue, red and black circles indicate the remembered positions of targets previously shown at the horizontal positions −6°, −2°, 2°, and 6°, respectively. All targets are placed at 5° vertical position. This shows that in the model, the magnitude of spatial updating is similar across different pursuit amplitudes / durations. **(B)** This panel shows the neural activity of an example neuron with receptive field centered at 5° right and 5° up relative to the gaze position. Simulated neural activity is plotted as a function of horizontal eye position corresponding to the trials in **(A)**. **(C)** This panel shows example eye movement trajectories of experimental data from a monkey, color coded for different lengths (Dash et al., 2015). **(D)** This shows the neural activity of an example neuron in SC for differently binned smooth pursuit lengths (corresponding to the examples above) and collapsed across different memory targets. Neural activities (obtained from multiple trials) are again color coded. This real example illustrates that spatial updating was also continuous for different pursuit magnitudes/durations, except that the SC neuron shows a slight decrement in the updating response over time/distance, which is not observed in our model. This is likely because the computational model was trained to provide ideal updating whereas the real brain has additional noise and synaptic imperfections in its feedback loops. For complete details of the experimental methods used to obtain the data in **(C,D)** see Dash et al. (2015).

Figure S2 **Effect of upcoming saccade on an example neuron activity, averaged across many trials. (A)** This diagram shows the averaged neural activity of a neuron with receptive field centered at (10° right, 5° up) relative to gaze position over 100 trials with the same configuration as presented in Figure 7A. As can be seen in this diagram, the neural activity (black spikes) raised before saccade onset. This shows a predictive remapping behavior of this neuron. **(B)** This diagram shows the averaged neural activity of a neuron with receptive field centered at (10° right, 5° up) relative to gaze position over 100 trials in the case the second target presented close to the saccade onset (similar to Duhamel et al., 1992 experiment). As can be seen in this diagram, similar to **(A)** the neural activity (black spikes) raised before saccade onset. This shows the predictive remapping behavior of this neuron, and shows that noise is filtered out over trials, in the same way it would for uncorrelated noise across neurons.
