# Supplementary material for: A State Space Model for Spatial Updating of Remembered Visual Targets during Eye Movements
Source: Front Syst Neurosci. 2016 May 12;10:39. doi: 10.3389/fnsys.2016.00039 (PMC4867689; doi:10.3389/fnsys.2016.00039)
Supplement: Supplementary file 1 [file Presentation1.pdf]

A state space model for spatial updating of remembered visual targets during eye movements

Yalda Mohsenzadeh [1], **Suryadeep Dash** [1,2], **and John Douglas Crawford** [1,3,*]
*Correspondence:
John Douglas Crawford
jdc@yorku.ca

1 SUPPLEMENTAL MATERIALS

In this appendix, we clarify some aspects of the proposed model in more detail that can be used to reproduce the presented results in the paper.

MODEL FORMALIZATION FOR 2D CASE

As we explained in the paper, we developed a non-linear state space model to study the dynamics of spatial updating of remembered visual targets across eye movements:

$$TR_{k+1} = f(TR_k, EC_k, w_k) + \nu_k, \tag{S1}$$

$$EP_k = -TR_k + TS + n_k \tag{S2}$$

where EC_k is a two dimensional vector of corollary discharge (efference copy signal) at time point k and TS is the two dimensional vector of the target position in the head frame of reference (This term is necessary to transform the hidden state TR_k which is in gaze center frame of reference to the head center frame of reference in Equation (S2) as measurements (eye position signal EP_k) are in the head reference frame. Moreover, as we explained in the paper the function f in the state transition equation (S1) is implemented through a RBF neural network. For the 2D case this function is defined as following,

$$TR^x_{k+1} = \sum_{i=1}^{P} w^c_{x_i} a_i(TR_k - EC_k) + \nu^x_k \tag{S3}$$

$$TR^y_{k+1} = \sum_{i=1}^{P} w^c_{y_i} a_i(TR_k - EC_k) + \nu^y_k \tag{S4}$$

where $w^c_{x_i}$ and $w^c_{y_i}$ for $i = 1, 2, ...P$ [1] are post-connection weights and their values are proportional to μ^x_i and μ^y_i, respectively. To elaborate more, remember that we can decode the peak of a Gaussian hill of

[1] Our model includes $P = 61^2$ neurons and as it will be explained in the following only a subset of these neurons are activated at each step (tentatively around 114 neurons).

activities by calculating the center of mass of this hill as following

$$x(t) = \frac{\sum_i \mu_i^x \phi_i(t)}{\sum_j \phi_j(t)} \tag{S5}$$

$$y(t) = \frac{\sum_i \mu_i^y \phi_i(t)}{\sum_j \phi_j(t)} \tag{S6}$$

We can rewrite the above equations as following:

$$x(t) = \sum_i \left(\frac{\mu_i^x}{\sum_j \phi_j(t)} \phi_i(t)\right) \simeq \sum_i \mu_i^x A_i(t)\phi_i(t) \tag{S7}$$

$$y(t) = \sum_i \left(\frac{\mu_i^y}{\sum_j \phi_j(t)} \phi_i(t)\right) \simeq \sum_i \mu_i^y A_i(t)\phi_i(t) \tag{S8}$$

Equations (S7) and (S8) explain the definition of Equations (S3) and (S4). Following the description presented in (S3) and (S4), the online estimation of parameters and memory target position follow the equations presented in Section 2.5. To elaborate more, the model performs the following steps recursively:

1. Target location prediction: The target location is predicted based on (S3) and (S4), i.e. $\overline{TR}_k = [TR_k^x \;\; TR_k^y]^t$ (Here t denotes transposition).
2. Target location correction using noisy measurements: The Kalman gain matrix is calculated based on the following equations:
$$KG_k = -\Sigma_k^{TR}(\Sigma_k^{TR} + \sigma_{EP}^2 I_2)^{-1} \tag{S9}$$
where
$$\Sigma_k^{TR} = (I_2 - KG_{k-1})(F_{k-1}\Sigma_{k-1}^{TR}F_{k-1}^T + \sigma_{TR}^2 I_2) \tag{S10}$$
where I_2 denotes 2×2 identity matrix and $F_k = \frac{\partial f}{\partial TR}|_{\widehat{TR}_k}$. Finally the predicted target location in step 1 is corrected based on the available eye position observation according to the following equation:
$$\widehat{TR}_k = \overline{TR}_k + KG_k(EP_k - (TS - \overline{TR}_k)) \tag{S11}$$
3. Checking for possible growing: In this step the neuron contribution criterion is calculated for each inactive neuron in the model according to Equation (16). If the neuron contribution satisfies the condition presented in (17) then the neuron is activated; i.e. $A_i = A_0$ and $\sigma_i = \sigma_0$. The threshold e_{min} depends on the task (saccade or pursuit) as well as other predefined parameters such as A_0, σ_0. For example for $A_0 = 1$, $\sigma_0 = 7$ and smooth pursuit $e_{min} = 0.0001$ and for saccade $e_{min} = 0.01$.
4. Model parameters prediction: Model parameters include A_i and σ_i for all activated neurons. The value for these parameters are easily estimated based on Equation (9) for next time step.
5. Model parameter correction: The predicted values for model parameters are corrected using Equations (11) to (13) where $C_k^w = \frac{\partial f}{\partial w}|\widehat{w}_k$.
6. Checking for possible pruning: After updating the model parameters which determine the neural activities, we check again for any possible pruning. In other words, we calculate the neuron contribution criterion for every activated neuron and if $E_{cont}(i) < e_{min}$, the ith neuron is deactivated; i.e. $A_i = 0$ and $\sigma_i = \sigma_0$.

The above steps are implemented recursively through time until the end of each trial. The matrices F_k and C_k^w in steps 2 and 5, respectively, are calculated as following.

$$F_k = \frac{\partial f(TR, w_k)}{\partial TR}|_{\widehat{TR}_k} \tag{S12}$$

where f is already defined in Equation (8) for 1D or (S3) and (S4) for 2D. For 1D, F_k is a scaler and calculated as:

$$F_k = -2\sum_{i=1}^{P} w_i^c \frac{(\widehat{TR}_k - \mu_i)}{\sigma_i^2} \exp\left(-\frac{(\widehat{TR}_k - \mu_i)^2}{\sigma_i^2}\right) \tag{S13}$$

In the 2D case, F_k is a 2×2 matrix and its elements are calculated as $F_k^{11} = \frac{\partial f(TR^x, w_k)}{\partial TR^x}|_{\widehat{TR^x}_k}$, $F_k^{12} = \frac{\partial f(TR^x, w_k)}{\partial TR^y}|_{\widehat{TR^y}_k}$, $F_k^{21} = \frac{\partial f(TR^y, w_k)}{\partial TR^x}|_{\widehat{TR^x}_k}$, and $F_k^{22} = \frac{\partial f(TR^y, w_k)}{\partial TR^y}|_{\widehat{TR^y}_k}$ following a similar procedure as presented in Equation (S13). Finally, C_k^w the linearization associated with the model parameters $\{A_i, \sigma_i\}_{i=1}^P$ as:

$$C_k^w = \frac{\partial TR}{\partial w}|_{\widehat{w}_k} \tag{S14}$$

where TR is defined in (8) for 1D or (S3) and (S4) for 2D. Therefore according to the chain rule we can write:

$$\frac{\partial TR}{\partial w} = \frac{\partial f(\widehat{TR}_k, w)}{\partial \widehat{TR}_k}\frac{\partial \widehat{TR}_k}{\partial w} + \frac{\partial f(\widehat{TR}_k, w)}{\partial w} \tag{S15}$$

and using (S11), we can write:

$$\frac{\partial \widehat{TR}_k}{\partial \widehat{w}} = (I - KG_k C)\frac{\partial f}{\partial \widehat{w}} + \frac{\partial KG_k}{\partial \widehat{w}}(EP_k - (TS - \widehat{TR}_k)) \tag{S16}$$

Finally, the terms $\frac{\partial f(\widehat{TR}, \widehat{w})}{\partial \widehat{TR}_k}$ and $\frac{\partial f(\widehat{TR}, \widehat{w})}{\partial \widehat{w}_k}$ can be calculated easily using the Equations ((8) for 1D or (S3) and (S4) for 2D [2]. We can assume the Kalman gain KG_k is independent of w. Then the last term in Equation (S16) will be dropped.

[2] Please note that for the case of 2D the Kalman gain and errors in x and y dimensions are calculated and the both terms are used for correction.
