# Supplementary figures and images for: A State Space Model for Spatial Updating of Remembered Visual Targets during Eye Movements
Source: Front Syst Neurosci. 2016 May 12;10:39. doi: 10.3389/fnsys.2016.00039 (PMC4867689; doi:10.3389/fnsys.2016.00039)

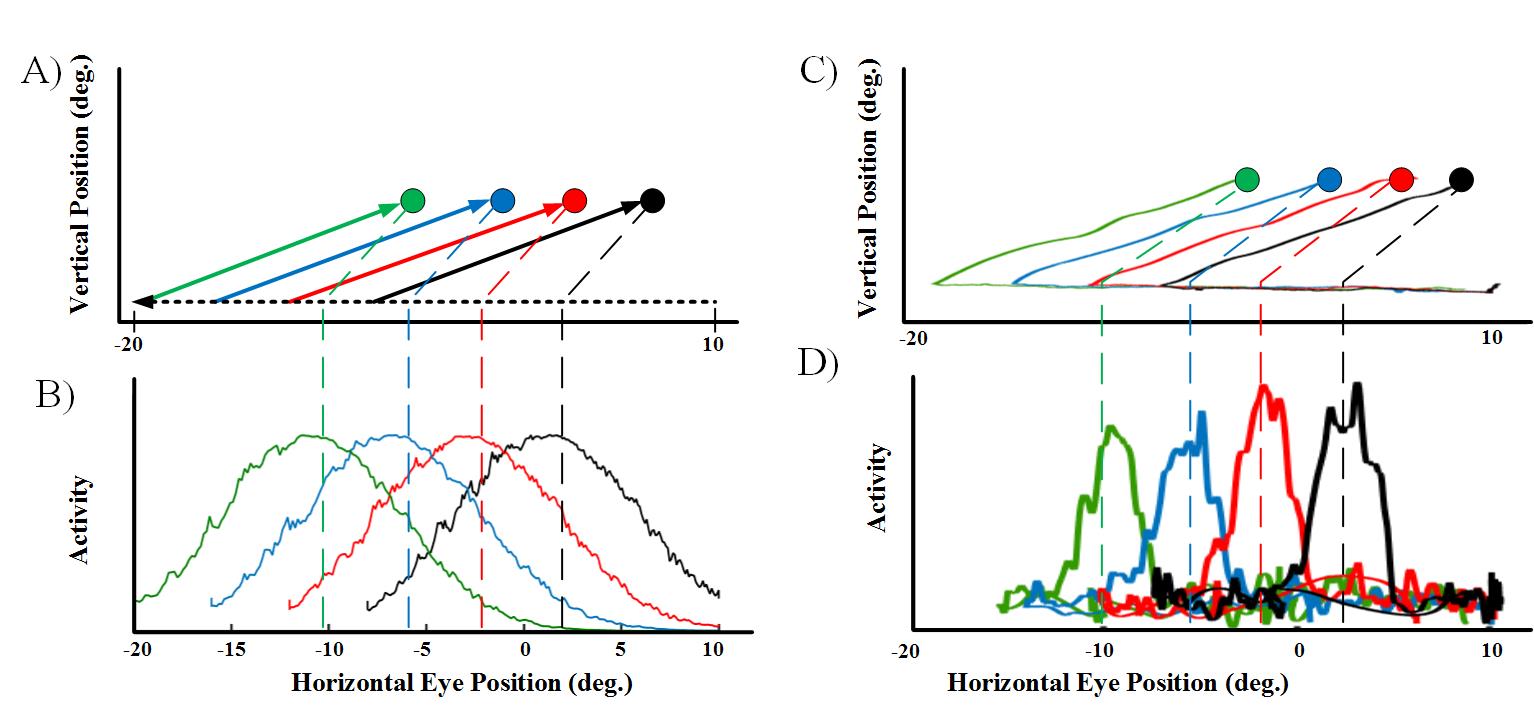

Supplement: Figure S1 — Spatial updating behavior for different smooth pursuit lengths/durations. (A) Eye movement trajectories in 2D spatial coordinates. This panel shows four leftward constant velocity smooth pursuit ramps (18°, 22°, 26°, and 30°) followed by a saccade to different previously shown visual targets. The green, blue, red and black circles indicate the remembered positions of targets previously shown at the horizontal positions −6°, −2°, 2°, and 6°, respectively. All targets are placed at 5° vertical position. This shows that in the model, the magnitude of spatial updating is similar across different pursuit amplitudes / durations. (B) This panel shows the neural activity of an example neuron with receptive field centered at 5° right and 5° up relative to the gaze position. Simulated neural activity is plotted as a function of horizontal eye position corresponding to the trials in (A). (C) This panel shows example eye movement trajectories of experimental data from a monkey, color coded for different lengths (Dash et al., 2015). (D) This shows the neural activity of an example neuron in SC for differently binned smooth pursuit lengths (corresponding to the examples above) and collapsed across different memory targets. Neural activities (obtained from multiple trials) are again color coded. This real example illustrates that spatial updating was also continuous for different pursuit magnitudes/durations, except that the SC neuron shows a slight decrement in the updating response over time/distance, which is not observed in our model. This is likely because the computational model was trained to provide ideal updating whereas the real brain has additional noise and synaptic imperfections in its feedback loops. For complete details of the experimental methods used to obtain the data in (C,D) see Dash et al. (2015). [file Image1.jpeg]

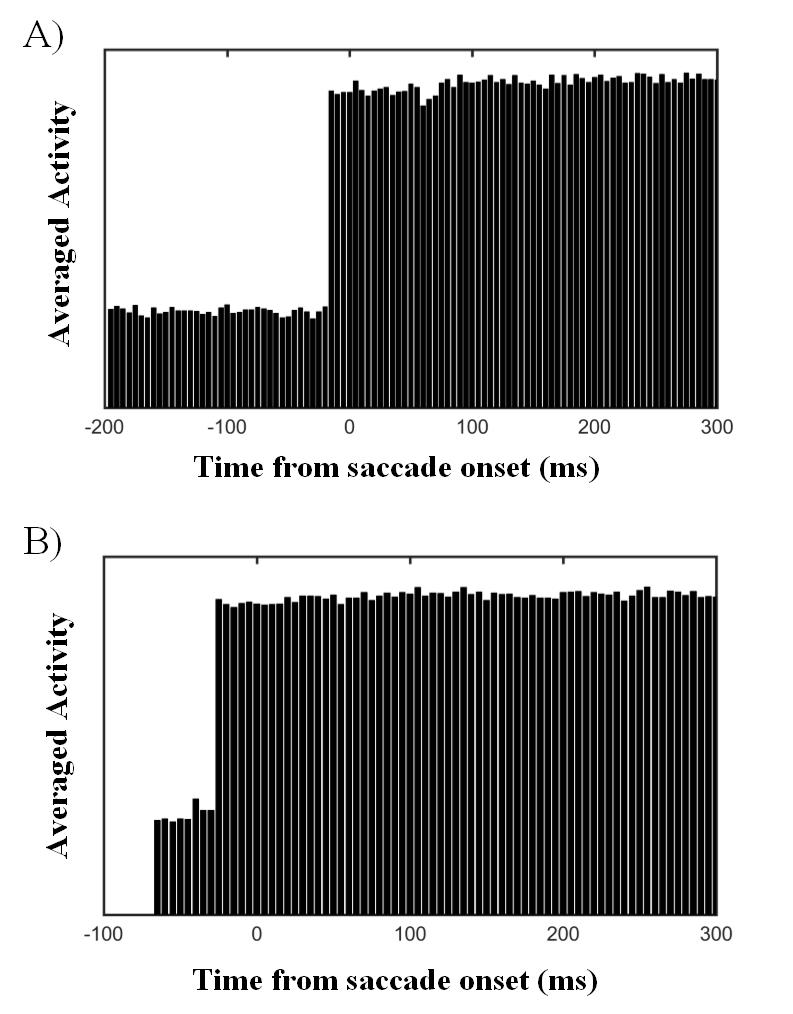

Supplement: Figure S2 — Effect of upcoming saccade on an example neuron activity, averaged across many trials. (A) This diagram shows the averaged neural activity of a neuron with receptive field centered at (10° right, 5° up) relative to gaze position over 100 trials with the same configuration as presented in Figure 7A. As can be seen in this diagram, the neural activity (black spikes) raised before saccade onset. This shows a predictive remapping behavior of this neuron. (B) This diagram shows the averaged neural activity of a neuron with receptive field centered at (10° right, 5° up) relative to gaze position over 100 trials in the case the second target presented close to the saccade onset (similar to Duhamel et al., 1992 experiment). As can be seen in this diagram, similar to (A) the neural activity (black spikes) raised before saccade onset. This shows the predictive remapping behavior of this neuron, and shows that noise is filtered out over trials, in the same way it would for uncorrelated noise across neurons. [file Image2.jpeg]
